# Analysis of transcript-deleterious variants in Mendelian disorders: implications for RNA-based diagnostics

**DOI:** 10.1186/s13059-020-02053-9

**Published:** 2020-06-17

**Authors:** Sateesh Maddirevula, Hiroyuki Kuwahara, Nour Ewida, Hanan E. Shamseldin, Nisha Patel, Fatema Alzahrani, Tarfa AlSheddi, Eman AlObeid, Mona Alenazi, Hessa S. Alsaif, Maha Alqahtani, Maha AlAli, Hatoon Al Ali, Rana Helaby, Niema Ibrahim, Firdous Abdulwahab, Mais Hashem, Nadine Hanna, Dorota Monies, Nada Derar, Afaf Alsagheir, Amal Alhashem, Badr Alsaleem, Hamoud Alhebbi, Sami Wali, Ramzan Umarov, Xin Gao, Fowzan S. Alkuraya

**Affiliations:** 1 Department of Genetics, King Faisal Specialist Hospital and Research Center, Riyadh, Saudi Arabia; 2 Computational Bioscience Research Center (CBRC), Computer, Electrical, and Mathematical Sciences and Engineering (CEMSE) Division, King Abdullah University of Science and Technology (KAUST), Thuwal, Saudi Arabia; 3 Département de génétique, AP-HP, Hôpital Bichat, Université de Paris, LVTS INSERM U1148, Paris, France; 4 Deparmtent of Medical Genetics, King Faisal Specialist Hospital and Research Center, Riyadh, Saudi Arabia; 5 Department of Pediatrics, King Faisal Specialist Hospital and Research Center, Riyadh, Saudi Arabia; 6 Department of Pediatrics, Prince Sultan Military Medical City, Riyadh, Saudi Arabia; 7 Department of Anatomy and Cell Biology, College of Medicine, Alfaisal University, Riyadh, Saudi Arabia; 8 Division of Pediatric Gastroenterology, Children’s Hospital, King Fahad Medical City, Riyadh, Saudi Arabia

**Keywords:** Negative WES, RNA-based diagnostics, Mapping, Mendelian, Transcriptomics

## Abstract

**Background:**

At least 50% of patients with suspected Mendelian disorders remain undiagnosed after whole-exome sequencing (WES), and the extent to which non-coding variants that are not captured by WES contribute to this fraction is unclear. Whole transcriptome sequencing is a promising supplement to WES, although empirical data on the contribution of RNA analysis to the diagnosis of Mendelian diseases on a large scale are scarce.

**Results:**

Here, we describe our experience with transcript-deleterious variants (TDVs) based on a cohort of 5647 families with suspected Mendelian diseases. We first interrogate all families for which the respective Mendelian phenotype could be mapped to a single locus to obtain an unbiased estimate of the contribution of TDVs at 18.9%. We examine the entire cohort and find that TDVs account for 15% of all “solved” cases. We compare the results of RT-PCR to in silico prediction. Definitive results from RT-PCR are obtained from blood-derived RNA for the overwhelming majority of variants (84.1%), and only a small minority (2.6%) fail analysis on all available RNA sources (blood-, skin fibroblast-, and urine renal epithelial cells-derived), which has important implications for the clinical application of RNA-seq. We also show that RNA analysis can establish the diagnosis in 13.5% of 155 patients who had received “negative” clinical WES reports. Finally, our data suggest a role for TDVs in modulating penetrance even in otherwise highly penetrant Mendelian disorders.

**Conclusions:**

Our results provide much needed empirical data for the impending implementation of diagnostic RNA-seq in conjunction with genome sequencing.

## Introduction

Genome sequencing, enabled by the advent of next-generation sequencing (NGS) technologies, has changed the landscape of diagnostics in the Mendelian diseases space [1]. Whole-exome sequencing (WES) is the most popular NGS diagnostic application and has achieved a diagnostic rate of 25–52% across the spectrum of Mendelian disorders, although higher figures have been reported for certain phenotypic categories [2–5]. The minimal boost of diagnostic yield offered by whole-genome sequencing (WGS) over WES suggests that the bottleneck is not in the capture/calling of the causal variants in the sequencing stage but rather in their interpretation [6, 7]. This notion is supported by studies showing the value of careful reinterpretation of “negative” WES and how misinterpreting the causal variants in WES is a major challenge that cannot be circumvented by WGS [7, 8]. Therefore, there is a growing interest in exploring transcriptomics to improve variant interpretation [9]. Indeed, published data suggest an enrichment of “negative” WES cases for cryptic splice-altering variants that are not easily predicted in silico [10, 11].

Coding genomic variants modulate phenotypes through their effect on proteins while non-coding variants (NCV) mediate their effects through RNA either directly (transcript-level) or indirectly (chromatin-level). In the context of Mendelian diseases, estimates vary widely on the contribution of variants that affect splicing to the overall mutation pool (15–60% of disease-causing variants) [12]. Two major challenges preclude accurate estimation of this important class of disease-causing mutations. First, many “coding” variants that are presumed to exert their pathogenicity at the protein level are in fact splicing variants whose effect on splicing was never empirically determined. These not only include single base-pair substitutions that may or may not alter the amino acid sequence (nonsynonymous and synonymous missense), but also include protein-truncating variants [13]. Another major challenge is the clear reporting bias in the literature where variants that impact consensus splicing codes are more likely to be tested and reported. Deep intronic, UTR and promoter/enhancer variants are far less likely to be uncovered by conventional Sanger or WES and, even when captured by WGS, are very difficult to interpret using in silico tools despite their clear contribution to Mendelian diseases [14–17].

Transcriptomics, therefore, holds a promising role in delineating Mendelian phenotypes that are caused by variants that are deleterious at the transcript level [18]. These include variants that reduce the abundance of the transcript, e.g., nonsense-mediated decay (NMD), as well as those that create aberrant splicing. Early experience with RNA-Seq (massively parallel sequencing of RNA) suggests its potential to reveal variants that have been missed at the sequencing stage as well as those that have been missed at the interpretation stage [10, 11, 19–21]. It is also clear from these studies, however, that there are unique computational challenges to this technology, and although several computational tools have been developed, there is a growing need for a deeper understanding of the nature of transcript-deleterious variants to inform better tools. We have previously shown in a pilot study the power of positional mapping as a tool that is agnostic to the underlying class of mutation to provide unbiased estimate of NCVs [8]. In this study, we provide based on comprehensive positional mapping of 5647 families with suspected Mendelian phenotypes a detailed overview of transcript-level deleterious variants and their contribution to Mendelian phenotypes in humans. We then interrogate the translational potential of that knowledge by exploring the role of RNA-based approaches in patients with “negative” clinical WES results.

## Materials and methods

### Human subjects

Subjects described in this study represent combined cohorts recruited under individual IRB-approved research protocols (KFSHRC RAC# 2121053, 2080006 and 2070023). In each of these protocols, we selectively recruited individuals with at least one of the following features: (a) positive family history consistent with a Mendelian inheritance of the disorder and (b) phenotypic presentation consistent with a previously published Mendelian disease. Informed consent was obtained from all subjects prior to their enrollment. Phenotypic data were collected from all subjects. Blood was collected in EDTA tubes for DNA extraction and in sodium heparin tubes for the establishment of lymphoblastoid cell lines (LCL). Occasionally, blood collected in PAXGene tubes was the only source of RNA. In a subset of cases, cultured skin-derived fibroblasts and urine-derived renal epithelial cells were also obtained as an additional source of RNA.

### Positional mapping, WES, and variant identification

The method of combining positional mapping and variant identification using WES has been described elsewhere [1, 22]. Briefly, all samples were genotyped on an Axiom SNP platform, and the regions of homozygosity (ROH) were determined to guide the search for the likely causal variant whenever the phenotype and family history are compatible with autosomal recessive inheritance. WES was performed as described before, and the resulting variants were filtered by the autozygome coordinates [3, 23]. Variants were filtered using gnomAD and a local population database (2379 exomes) for allele frequency of < 0.001 and were interpreted by following the ACMG guidelines [24] to determine the likely causal variants. Although protein-truncating variants may exert their pathogenic effect at the level of the final transcript via NMD, we have chosen to exclude them because it is very difficult to disentangle their effect on protein from that on RNA. Variants were highlighted as candidate transcript-deleterious variants (TDVs) if they were compatible with pathogenicity potential in terms of frequency and segregation, and involved one of the following six categories: (a) canonical splice donor or acceptor sites (the first and last 2 bp of each intron), (b) the first or last base pair of an exon, (c) non-canonical splice site intronic variants, i.e., other than the first and last 2 bp of an intron, (d) coding exons other than the first or last base pair (regardless of whether the resulting missense is synonymous or nonsynonymous), (e) UTR (5′ and 3′), and (f) promoter/enhancer elements. Variants in categories c, d, e, and f were only considered if no alternate candidate variants were identified. A small subset of cases for which no candidate variants were identified, were subjected to RNA-Seq (see below).

### RTPCR

Variants suspected to be deleterious at the transcript level were interrogated by RTPCR using cDNA-specific primers and RNA from blood (LCL or PAXgene) and/or skin fibroblasts. When the index who is homozygous for variant was unavailable, we attempted to test the obligate heterozygous parents. RTPCR followed a standard number of 35 cycles and 2000 ng of RNA as a template. If this standard protocol resulted in a visible band on a gel, the gene was considered “expressed.” If additional cycles or higher amount of RNA were needed, the gene was considered “poorly expressed,” otherwise, the gene was labeled as “not expressed.” The products were analyzed by Sanger sequencing directly and if there was evidence of multiple products, cloning was pursued followed by Sanger sequencing. In cases where no evidence of aberrant splicing was identified, we attempted quantifying the transcript using q-RTPCR.

### RNA-Seq and computational analysis

RNA samples of the subjects were prepared at KFSHRC and sent to the KAUST core lab for RNA sequencing. The quality of each RNA sample was determined based on its RNA Integrity Number (RIN) using Agilent 2100 BioAnalyzer. Those samples that scored RIN < 6.0 were not considered further. The sequencing libraries were prepared using Illumina TruSeq Stranded mRNA. Paired-end 150 bp reads were generated on Illumina NovaSeq6000. GTEx RNA-Seq samples [25] for blood and skin tissue types were downloaded from the Database of Genotypes and Phenotypes (dbGaP) and transformed into the fastq format using SRA Toolkit (https://www.ncbi.nlm.nih.gov/sra/docs/toolkitsoft/). Samples with RIN < 8.0 were not included in our GTEx controls. RNA-Seq reads from both patients and GTEx were also aligned to hg38 (GENCODE 25) using STAR 2.6 [26] with the two-pass option. Only reads mapped to chromosomes 1–22 and X were considered. SAMtools [27] and BEDTools [28] were applied to the BAM files to quantify the occurrence of annotated and unannotated splicing junctions, as well as to count nonsplit reads mapped to intronic regions. Splicing junctions with < 5 read supports were filtered out. To quantify the transcript abundance levels, RNA-Seq reads were also mapped to the reference transcript sequences for hg38 (GENCODE 25) using Kallisto [29]. Using the generated BAM files and transcript abundance levels, error-free normal transcript abundance levels were estimated with the omega quantification [30]. Briefly, omega computes an adjusted count per million (CPM) value for each coding gene *g, ω*_*g*_*,* as follows:

$$ {\omega}_g=\sum \limits_{t\in {T}_g}{w}_t{x}_t $$

where *T*_*g*_ is the set of mRNA transcripts for gene *g*, *w*_*t*_ is the rate to express annotated, normal transcript *t* based on the RNA splicing data, and *x*_*t*_ is the CPM level of transcript *t*. Thus, low values of *ω*_*g*_ can indicate low abundance outliers or splicing outliers that escaped NMD.

From the GTEx data, RNA-Seq datasets of the "Cells - EBV-transformed lymphocytes" and "Cells - Cultured fibroblasts" tissue types were selected as the control for cases derived from blood and skin tissue types, respectively. To ensure the use of an appropriate set of samples in control for each patient, we measured the median of the *ω*_*g*_ values of each coding gene for all the blood and skin tissue types in the GTEx datasets and confirmed that the selected tissue type gave the highest level of correlation with the patient data.

Based on the second percentile of the *ω*_*g*_ values in the corresponding control, two scores, *α*_*g*_ and *β*_*g*_, were measured to analyze the severity of transcriptional aberrations in gene *g* for each patient. Let *ω*_*g*_ (*i*) and *ω*_*g*_ (*k*_*i*_) represent the value of *ω*_*g*_ for patient *i* and for the second percentile value of the corresponding control *k*_*i*_, respectively. Then, score *α*_*g*_(*i*) was computed as follows:

$$ {\alpha}_g(i)=\frac{\omega_g\left({k}_i\right)}{\omega_g(i)+\varepsilon } $$

where *ε* is a small factor set to 0.001 to avoid division by zero. The *α* score measures the significance of genes as the low abundance outliers or splicing outliers. The other score, *β*_*g*_, was derived by first computing the fraction of normal transcripts, *ρ*_*g*_ which is defined to be the ratio of *ω*_*g*_ to $\sum \limits_{t\in {T}_g}{x}_t.$ Given this definition, score *β*_*g*_(*i*) for patient *i* can be expressed as:

$$ {\beta}_g(i)=\frac{\rho_g\left({k}_i\right)}{\rho_g(i)+\varepsilon } $$

Thus, the *β* score measures the significance of likeliness to express transcripts with splicing error. A high alpha score means that the abundance level of normal transcripts of a given gene is lower compared with a lower-end abundance of the same gene in the control set. Similarly, a high beta score means that the fraction of the normal transcripts of a given gene is lower compared with a lower end of the same gene in the control set. With these scores, each coding gene *g* was selected as a causative candidate for each patient *i* if all of the following criteria are met:

1. Either *α*_*g*_(*i*) ≥ 3.0 or *β*_*g*_(*i*) ≥ 3.0.
2. For all the other patients *j* with RNA samples being the same cell type, *α*_*g*_(*i*) < *α*_*g*_(*j*).

Note that criterion 2 is based on 11 RNA-Seq datasets from RNA samples with RIN > 8.5 (4 from fibroblasts and 7 from LCL) and that this criterion was set specifically for comparison based on a small number of patients. To visualize splicing events, BAM files were first converted into the hg19 coordinate using CrossMap [31] and Integrative Genomic Viewer [32] was used. We have also attempted to compare our method to previously published methods as explained in Additional file 1: Supplemental file 1.

## Results

### Quantifying the contribution of transcript-deleterious variants

**Fig. 1:**
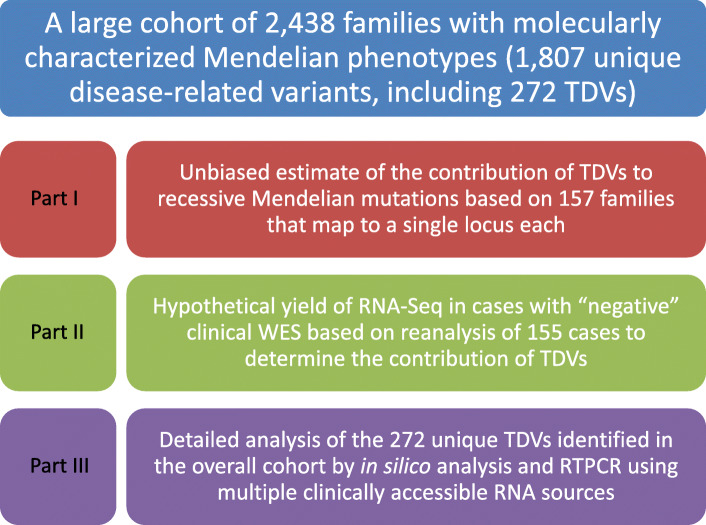
A flow chart of the entire study with its different components

**Fig. 2:**
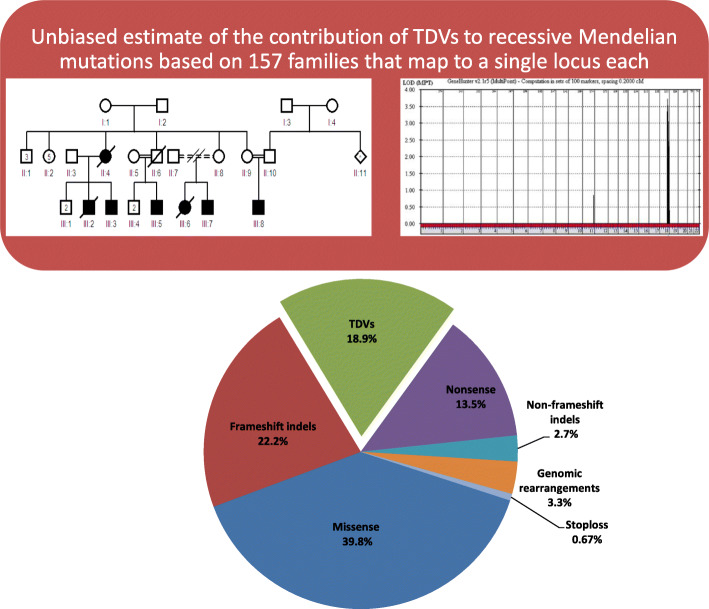
Unbiased estimate of the contribution of TDVs to recessive Mendelian mutations based on 157 families that map to a single locus each

Our cohort included 5647 families with suspected Mendelian phenotypes (Fig. 1). The vast majority (94% and 91%) are consanguineous and multiplex, i.e., > 1 affected member, respectively. A likely causal variant was identified in 2438 of these families (*n* = 1807 non-redundant variants), 272 (15%) of which represent TDVs (TDVs are listed in Additional file 2: Table S1, and their population frequencies are summarized in Additional file 3: Table S2). One limitation of this estimate is the potential for bias against the identification of more challenging classes of transcript-deleterious variants. Therefore, we decided to exploit the agnostic nature of positional mapping to derive unbiased estimate of transcript-deleterious variants. We singled out all families in which we were able to map their recessive Mendelian phenotype to a single locus (*n* = 157) since these lend themselves more readily to focused and thorough investigations to reveal the underlying variant including the most challenging ones. Indeed, each of these loci was thoroughly interrogated and this resulted in the identification of a likely causal variant (*n* = 148, two variants were observed in four cases) in 95.5% of cases (150 out of 157, Fig. 2 and Additional file 4: Table S3). The breakdown of variant classes within these loci shows that TDVs accounted for 18.9% of variants (28 out of 148), which suggests that the above figure of 15% may indeed represent an underestimate based on bias against more challenging transcript-deleterious variants. Interestingly, only 2% of the 18.9% are variants not expected to be captured by WES (> 50 bp from the nearest exon), which suggests that, at least in the case of recessive phenotypes in consanguineous families, the overwhelming majority of causal variants are captured by WES pipelines.

### RNA as a tool to solve “negative” WES cases

**Fig. 3:**
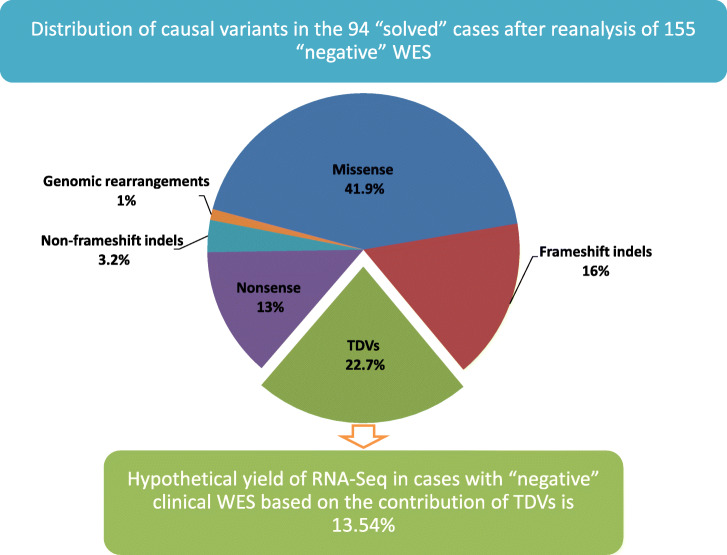
The results of our reanalysis of “WES-negative” cases to estimate the hypothetical yield of RNA-Seq in this clinical setting

In order to investigate the contribution of RNA analysis to solving “negative” WES cases, we recruited 155 cases for which clinical WES did not reveal a likely causal variant (Fig. 3, Table 1 and Additional file 5: Table S4). A likely causal variant was subsequently identified in 60.6% (88 unique variants in 94 out of 155 cases). Additional file 5: Table S4 shows that many of these cases harbored a likely deleterious variant in a gene that was novel at the time of clinical reporting, i.e., cases unlikely to have benefited from RNA analysis. TDVs accounted for 22.7% of all identified variants (20 out 88 unique variants). As expected, class (a) variants (those affecting the canonical splice sites) were under-represented (21% vs. 64% in the original cohort, see below) since these would have been readily flagged at the time of reporting. On the other hand, the more challenging classes were over-represented (79% vs. 36% in the original cohort, see below). These include a very deep (+ 335) variant in *ABCB4* causing cholestatic disease in all available affected members of an extended family (see Additional file 6: Figure S1). Thus, the hypothetical diagnostic yield of RNA-Seq in the setting of a “negative” WES is 13.5% (21 out 155 cases), at least in the setting of recessive phenotypes. To test this empirically, we set out to investigate the six cases (no RNA was available from the seventh case) whose autosomal recessive Mendelian phenotypes map to single loci, and have “negative” WES, using RNASeq. First, we aimed to establish the sensitivity of our RNA-Seq pipeline and comparing it to previously published pipelines by testing five cases with established transcript-deleterious variants and found that 100% were correctly called, i.e., the mutated gene was chosen among the top or only candidate gene for each of the five cases (Additional file 6: Figure S2 and Additional file 7: Table S5) as follows:

- 10DG0840 (a case of Troyer syndrome and a class (d) variant in *SPG20*, see Additional file 2: Table S1): The RNA-Seq-based prediction generated 167 candidates. Among them, *SPG20* was ranked 157th on the alpha score and 25th on the beta score. With the autozygome coordinate-based filtering, *SPG20* was found to be the only candidate.
- 11DG0165 (a case of congenital muscular dystrophy and a class (c) variant in *POMT2*, see Additional file 2: Table S1): The RNA-Seq-based prediction generated 195 candidates. Among them, *POMT2* was ranked 2nd on the alpha score and 13th on the beta score. With the autozygome coordinate-based filtering, *POMT2* was found to be the top among the 14 final candidates.
- 15DG2154 (a case of microcephalic primordial dwarfism and a class (c) variant in *DONSON*, see Additional file 2: Table S1): The RNA-Seq-based prediction generated 324 candidates. Among them, *DONSON* was ranked 200th on the alpha score and 281st on the beta score. With the autozygome coordinate-based filtering, *DONSON* was found to be the only candidate.
- 16DG1048 (a case of peroxisomal disorder and a class (d) variant in *PEX19*, see Additional file 2: Table S1): The RNA-Seq-based prediction generated 129 candidates. Among them, *PEX19* was ranked 17th on the alpha score and 120th on the beta score. With the autozygome coordinate-based filtering, *PEX19* was found to be the only candidate.
- 16DG1620 (a case of osteopetrosis and a class (c) variant in *CLCN7*, see Additional file 2: Table S1): The RNA-Seq-based prediction generated 112 candidates. Among them, *CLCN7* was ranked 79th on the alpha score and 42nd on the beta score. With the autozygome coordinate-based filtering, *CLCN7* was found to be the top of the three remaining candidates.

**Table 1:** Summary of the negative clinical WES cases and genetic findings. For a full list of cases including negative and previously published, please refer to Additional file 5: Table S4. The symbol “a” indicates a novel gene (no assigned OMIM phenotype) for condition to be reported elsewhere

ID	Phenotype	Gene	Mutation HGVS nomenclature	Zygosity	Type of mutation	Class of transcript-deleterious variants	Notes
17DG0527	Global developmental delay, hypotonia, epilepsy, postnatal microcephaly, strabismus and choreoathetosis	*a*		Homozygous	Frameshift indel		Novel gene for this condition
15DG1507	Epilepsy and global developmental delay	*a*		Homozygous	Transcript-deleterious variant	c	Novel gene for this condition
18DG0320	Multiple congenital anomalies	*a*		Homozygous	Missense		Novel gene for this condition
18DG0989	Neonatal adrenoleukodystrphy	*a*		Homozygous	Missense		Novel gene for this condition
19DG0509	Undefined epileptic encephalopathy	*a*		Homozygous	Nonsense		Novel gene for this condition
18DG0669	Microcephaly, atrial septal defect, ventricular septal defect	*a*		Homozygous	Transcript-deleterious variant	a	Novel gene for this condition
17DG0738	Chronic interstitial kidney disease with small kidneys	*a*		Homozygous	Nonsense		Novel gene for this condition
19DG0230	Griscelli syndrome	*a*		Homozygous	Missense		Unpublished
17DG0872	Hydrocephalus, agenesis of corpus callosum, macrocephaly	*a*		Homozygous	Transcript-deleterious variant	b	Novel gene for this condition
17DG1071	Cholestasis, progressive familial intrahepatic 3	*ABCB4*	NM_000443.3:c.286+335A>G:p.(Val96Glyfs*11)	Homozygous	Transcript-deleterious variant	c	Unpublished
16DG0145	Intellectual disability	*ADAT3*	NM_138422.1:c.382G>A:p. (Val128Met)	Homozygous	Missense		Unpublished
16DG1223	Oligohydramios, short long bones and echogenic kidneys	*ANKS3*	NM_133450:c.352G>A:p.(Ala118Thr)	Homozygous	Missense		PMID:27417436
18DG0295	Joubert syndrome	*ARL3*	NM_004311.3:c.445C>T:p.(Arg149Cys)	Homozygous	Missense		PMID: 30269812
15DG2104	Joubert Syndrome	*ARMC9*	NM_025139.3:c.51+5G>T:p.?, r.1_51del	Homozygous	Transcript-deleterious variant	c	PMID: 27431290
15DG2485	Asparagine synthetase deficiency	*ASNS*	NM_133436.2:c.28A>C:p.(Ser10Arg)	Homozygous	Missense		PMID:30214071
15DG0357	Bardet-Biedl syndrome	*BBS1*	NM_024649.4:r. [1232_3423del]	Homozygous	Large deletion		PMID: 27894351
16DG1620	Osteopetrosis	*CLCN7*	NM_001287.5:c.739-18G>A;(p.Met250Argfs*6)	Homozygous	Transcript-deleterious variant	c	PMID: 29620724
19DG1262	Multiple congenital anomalies	*COG6*	NM_020751.2:c.695-8T>G	Homozygous	Transcript-deleterious variant	c	Unpublished
PSMMC0118	Short stature on growth hormone replacement, subclinical hypothyroidism, grade 1 hydronephrosis (Lt), delayed bone age, IVF pregnancy, first of a twin, delayed development	*CREBRF*	NM_001168393.2:c.475delT;p.(Ser159Hisfs*57)	Heterozygous	Frameshift indel		PMID: 31130284
17DG0967	Cholestasis with high GGT and renal failure	*DCDC2*	NM_001195610.1:c.223_293del:p.(Arg75Leufs*16)	Homozygous	Frameshift indel		Unpublished
17DG0996	Cholestasis	*DCDC2*	NM_001195610.1:c.223_293del:p.(Arg75Leufs*16)	Homozygous	Frameshift indel		Unpublished
13DG2237	Warsaw breakage syndrome	*DDX11*	NM_004399.2: c.2426T>G:p. (Val809Gly)	Homozygous	Missense		PMID: 30214071
17DG0022	Chronic unexplained diarrhea	*DGAT1*	NM_012079.5:c.836T>C:p.(Leu279Pro)	Homozygous	Missense		Unpublished
16DG0357	Acromesomelia	*DIP2C*	NM_014974.2:c.3283C>T:p.(Arg1095Trp)	Homozygous	Missense		PMID:29620724
17DG0756	Congenital disorder of glycosylation	*FUT8*	NM_178155.2:c.943C>T:p.(Arg315*)	Homozygous	Nonsense		PMID: 30237576
16DG0733	Severe progressive microcephaly, global developmental delay and epilepsy	*GPR56*	NM_005682.5:c.1503C>A;p.(Tyr501*)	Homozygous	Nonsense		PMID: 27431290
PSMMC0115	Unexplained macrocephaly, epilepsy, short stature and developmental delay	*KCND1*	NM_004979.6:c.1883G>A:p.(Arg628Lys)	Hemizygous	Missense		Unpublished
15DG2234	Microcephaly, cerebral white matter abnormality and intellectual disability	*KCTD3*	NM_016121.3 c.1036_1073del:p.(P346Tfs*4)	Homozygous	Transcript-deleterious variant	d	Unpublished
13DG2107	Psychomotor retardation and seizures	*KCTD3*	NM_016121.3 c.1036_1073del:p.(P346Tfs*4)	Homozygous	Transcript-deleterious variant	d	PMID:25558065
17DG0404	High GGT neonatal cholestasis/sclerosing cholangitis	*KIF12*	NM_138424.1:c.610G>A:p.(Val204Met)	Homozygous	Missense		PMID: 30250217
18DG0966	Methylmalonic aciduria and homocystinuria	*LMBRD1*	NM_018368.4:c.1156C>T:p.(Arg386*)	Homozygous	Nonsense		Unpublished
16DG0559	Joubert syndrome	*LRRC34*	NM_001172779:c.199A>T:p.(Lys67*)	Homozygous	Nonsense		In press
17DG0731	Disseminated tuberculosis, hypogammaglobulinemia, nearly all T and B cells are naive	*MAP3K14/NIK*	NM_003954.3:c.916delT: p.(Cys306Valfs*2)	Homozygous	Frameshift indel		doi.org/10.1016/j.jaci.2018.11.003
15DG2492	Short stature, global developmental delay, dysmorphism, congenital heart disease , PUJ obstruction and partial agenesis of corpus callosum	*MFSD11*	NM_001242532.1:c.143G>C:p.(Gly48Ala)	Homozygous	Missense		PMID: 28940097
16DG0621	Severe neurodevelopmental disorder	*MICU2*	NM_152726.3:c.42G>A:p.(Trp14*)	Homozygous	Nonsense		PMID: 29053821
17DG1094	Megacystis	*MYH11*	NM_022844.2:c.1033+1G>A	Homozygous	Transcript-deleterious variant	a	PMID: 30237576
12DG2078	Klippel-Feil syndrome and myopathy	*MYO18B*	NM_032608.5:c.6905C A:p.(Ser2302*)	Homozygous	Nonsense		PMID:25748484
18DG0176	Microcephaly, developmental delay, visual impairment, hyponatremia, failure to thrive, choreoathetoid movement, seizures	*NUP214*	NM_005085:c.461:p.(Asp154Gly)	Homozygous	Missense		PMID:30758658
16DG1424	Diarrhea, failure to thrive, intestinal failure and TPN dependence	*PERCC1*	Deletion of regulatory element (chr16:1480850_1483950del)	Homozygous	Transcript-deleterious variant	f	Unpublished
18DG0670	Erythrokeratoderma	*PERP*	NM_022121.4:c.466G>A:p.(Gly156Arg)	Homozygous	Missense		PMID: 31898316
16DG1048	Peroxisome biogenesis disorder 12A (Zellweger)	*PEX19*	NM_001193644.1:c.161C>T:p. (Ser54Leu)	Homozygous	Missense		PMID: 30561787
13DG0810	Congenital Microcephaly	*PPFIBP1*	NM_001198915.1:c.960_961del:p.(Glu320Aspfs*3	Homozygous	Frameshift indel		PMID: 30214071
16DG0201	Short stature, brachydactyly, intellectual disability and seizures	*PRMT7*	NM_019023.2:c. 190C>T:P. (Gln64*)	Homozygous	Nonsense		PMID: 28940097
15DG2427	Syndromic cataract	*RIC1*	NM_020829.3: c.3794G>C:p.(Arg1265Pro)	Homozygous	Transcript-deleterious variant	b	PMID: 27878435
13DG1181	Primary microcephaly	*RTTN*	NM_173630.3:c.5746-20A>G:p.1917_1942del	Homozygous	Transcript-deleterious variant	c	PMID: 30214071
17DG1005	Bardet-Biedl syndrome	*SCLT1*	NM_144643.2:c.290+2T>C:p.(Lys79Valfs*4)	Homozygous	Transcript-deleterious variant	a	PMID: 30237576
16DG0760	Epilepsy, generalized, with febrile seizures plus, type 1	*SCN1B*	NM_001037.3:c.355T>G:p.(Tyr119Asp)	Homozygous	Missense		PMID: 28218389
PSMMC0210	Hypotonia, global developmental delay, cardiac disease, leukodystophy	*SCN3A*	NM_001081676.1:c.1485T>G:p.(Ser495Arg)	Heterozygous	Missense		Unpublished
18DG0278	Congenital insensitivity to pain	*SCN9A*	NM_002977.3:c.2311-14T>G	Homozygous	Transcript-deleterious variant	c	Unpublished
14DG0045	Renal failure, morbid obesity, intellectual disability, retinitis pigmentosa (sibling of 14DG0047, see Table S5)	*SDCCAG8*	NM_006642.2:c.741-152G>A, p.Arg247Serfs*23; NM_006642.2: r.740_741ins741-202_741-1	Homozygous	Transcript-deleterious variant	c	In press
16DG0276	Tricho-Hepato-Enteric Syndrome	*SKIV2L*	NM_006929.5:c.3561_3581del; p.(Ser1189_Leu1195del)	Homozygous	Non-frameshift indel		Unpublished
16DG0815	Tricho-Hepato-Enteric Syndrome	*SKIV2L*	NM_006929.5:c.3561_3581del; p.(Ser1189_Leu1195del)	Homozygous	Non-frameshift indel		Unpublished
17DG0977	Tricho-Hepato-Enteric Syndrome	*SKIV2L*	NM_006929.4:c.3561_3581del,p.(Ser1189_Leu1195del)	Homozygous	Non-frameshift indel		Unpublished
18DG0594	Pseudovaginal perineoscrotal hypospadias	*SRD5A2*	NM_000348:c.682G>A:p.(Ala228Thr)	Homozygous	Missense		Unpublished
17DG0821	Congenital adrenal hyperplasia (CAH)	*STAR*	NM_000349.2:c.201_202del:p.(Tyr68Glnfs*2)	Homozygous	Frameshift indel		Unpublished
18DG0512	Osteogenesis imperfecta, type XIV	*TMEM38B*	NM_018112.2:c.455_542del;p.(Gly152Alafs*5)	Homozygous	Frameshift indel		Unpublished
16DG0114	Muscular dystrophy-dystroglycanopathy	*TMEM5*	NM_014254.3:c.686A>G:p.(Tyr229Cys)	Homozygous	Missense		Unpublished
16DG1117	Leukodystrophy	*TRAK1*	NM_001042646: c.287-2A>G	Homozygous	Transcript-deleterious variant	a	PMID:28940097
16DG0659	Muscular dystrophy, limb-girdle, autosomal recessive 18	*TRAPPC11*	NM_021942.6:c.464C>T:p.(Ser155Leu)	Homozygous	Missense		Unpublished
16DG1614	Global developmental delay and epilepsy	*UFC1*	NM_016406.3:c.317C>T:p. (Thr106Ile)	Homozygous	Missense		PMID: 29868776
16DG0018	Osteogenesis imperfecta	*WNT3A*	NM_033131.3:c.254G>A:p.(Arg85Gln)	Homozygous	Missense		PMID: 29620724
14DG0613	Primary microcephaly	*YARS*	NM_003680.3:c.789C>A:p.(Phe263Leu)	Homozygous	Missense		PMID: 28383543/30214071
15DG2661	Dysmorphism	*ZFAT*	NM_020863.3:c.1199G>A:p.(Arg400Gln)	Homozygous	Missense		PMID: 28640246/28940097

To test this empirically, we set out to investigate six cases whose autosomal recessive Mendelian phenotypes map to single loci with “negative” WES and for whom RNA sources were available. While no likely causal variant was identified in five of these cases, RNA-Seq analysis of blood-derived RNA on patient 15DG2234 (microcephaly, abnormality of the cerebral white matter and intellectual disability) highlighted *KCTD3* as the only likely candidate within the candidate autozygome (139 candidates were highlighted prior to the autozygome filter). Indeed, subsequent RTPCR confirmed that this pattern was created by a partial exonic deletion of 38 bps (NM_016121.3:c.1036_1073del:p.(Pro346Thrfs*4)) that was missed by WES and led to the creation of an additional aberrant band in which the involved exon was completely skipped (Additional file 6: Figure S2).

### The landscape of transcript-deleterious variants in Mendelian diseases

**Fig. 4:**
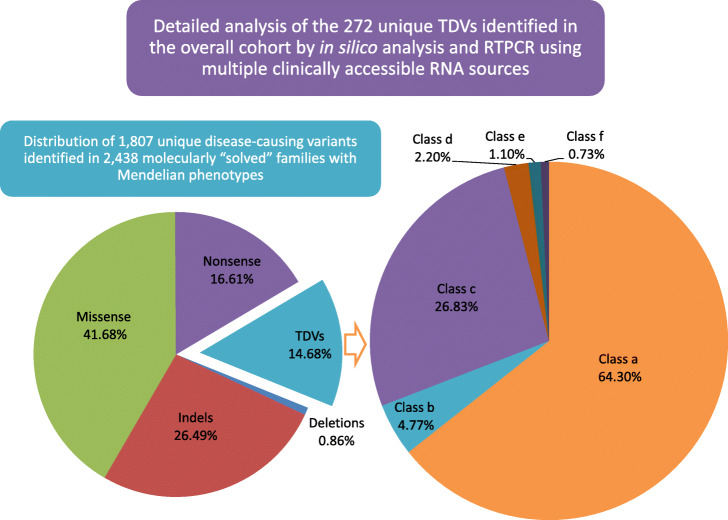
(Left) Pie chart showing the breakdown of variant types in a large cohort of families with suspected Mendelian disorders. (Right) Pie chart showing the distribution of all identified transcript-deleterious variants identified across the entire cohort. Classes a, b, c, d, e, and f represent the first or last 2 bp of introns, the first or last 1 bp of exons, non-canonical splice site intronic variants, non-canonical splice site exonic variants, UTR (5′ and 3′), and promoter variants, respectively.

Additional file 2 Table S1 lists all likely causal TDVs (272 unique variants) identified through our detailed analysis of 5647 families with suspected Mendelian phenotypes. The breakdown of the six classes of TDVs is summarized in Fig. 4 and is described below in detail.

1. Class (a) variants: a total of 175 (representing 64.3% of all TDVs) unique variants involving the canonical intronic splice sites were identified. RTPCR data were available for 93 (4 from literature and 89 from this cohort). Although this class is generally classified as “loss of function,” we note that several resulted in in-frame rather than frameshift indel (Additional file 2: Table S1). More concerning was the finding of canonical splicing variants in established disease genes with no resulting phenotype, i.e., non-penetrance (Additional file 8: Table S6). For example, the variant NM_001172818.1:c.300 + 1G > A in *PGM1*, was identified in homozygosity in individuals with no phenotype despite its deleterious effect on splicing (confirmed by RTPCR), which explains its high population frequency. Similarly, we have identified an individual with ambiguous genitalia who is homozygous for *LRP4* (NM_002334.2:c.796+2T>C) but lacks all features of established *LRP4*-related syndromes. On the other hand, the finding of *ARHGAP31* (NM_020754.2:c.539+1G>A) in asymptomatic individuals despite its deleterious effect on splicing (confirmed by RTPCR) can be attributed to the fact that previously reported mutations in this gene were proposed to be gain-of-function. The *SBDS* founder variant (NM_016038.2:c.258+2T>C) is also worth highlighting since this is the most commonly reported variant in Schwachman-Diamond syndrome (SDS) and yet we identified it in homozygosity in at least three individuals who lack SDS features. Upon further investigation, we found that this is a leaky splicing variant and that all previously reported SDS patients were compound heterozygous for a more severe truncating variant (Additional file 8: Table S6). Finally, we note the unusual result of normal RTPCR on a patient with Marfan syndrome and a de novo *FBN1 *(NM_000138.4:c.6872-1G>A) variant, which suggests that the effect of splicing may be tissue-specific (Additional file 8: Table S6).
2. Class (b) variants: RNA was available for 11 of the 13 variants involving the first or last bp of an exon, and in each of these cases an aberrant transcript was observed. This includes a variant in *TMX2* in case 19DG2556 with microcephaly and lissencephaly, which represents an independent confirmation of the very recently described *TMX2*-related disorder [33]. We suggest that this class should be combined with class (a) as canonical splice site variants. This is further supported by the consistently pathogenic prediction these variants received in silico (see below).
3. Class (c) variants: The range of non-canonical splice site intronic variants was remarkable ranging from 3 bp to 649 bp deep in our cohort. Since current capture techniques in WES usually capture < 50 bp of the flanking intronic sequence, we divided class c variants into those amenable for capture by WES, i.e., within 50 bp (*n* = 65) and those that are not, i.e., more than 50 bp from the nearest exon/intron junction (*n* = 8). The challenging nature of these variants is amplified when the phenotype is atypical (Additional file 8: Table S6). For instance, the NM_020751.2:c.1167-24A > G and NM_020751.2:c.695-8 T > G variants in *COG6* resulted in a phenotype sufficiently different from CDG that it is listed in OMIM as a separate disorder, i.e., Shaheen syndrome [34]. Similarly, we note the surprising finding of NM_182894.2:c.456-6C > G variant in *VSX2* causing ectopia lentis rather than the established microphthalmia phenotype, which supports a previously published case report [35]. Perhaps most surprising was the finding of a *homozygous NF1* variant (NM_001128147.2:c.586 + 5G>A) in a young child with juvenile myelomonocytic leukemia but the parents did not have any manifestations of neurofibromatosis (Additional file 8: Table S6). In an example of the challenge in proving the pathogenicity of this class of variants, we note that the previously published *COL6A2* (NM_001849.3:c.1459-63G>A) variant, which fully segregated with the expected phenotype of Ullrich muscular dystrophy, did not show abnormal RTPCR pattern suggesting the possibility of a tissue-specific splicing effect.
4. Class (d) variants: A total of 6 (2 exonic variants (excluding the first and last bp) were tested by RTPCR and found to be indeed transcript-deleterious. These include 3 that predict silent changes at the protein level.
5. Class (e) variants: Only three UTR (1.1%) variants were identified in the entire cohort (two 3′ UTR and one 5′ UTR mutation), suggesting their rarity, which is further supported by our unbiased analysis of families that map to single loci (Additional file 2: Table S1).
6. Class (f) variants: Only two variants (0.73%) were identified in the promoter or other regulatory regions of genes. The first is a TATA box mutation in *UGT1A1* (NM_000463.2:c.-41_-40dupTA) [36]. The second is a deletion (chr16:1480850_1483950del) in a patient with unexplained diarrhea, and this deletion was reported very recently to be the cause of chronic diarrhea secondary to its regulatory effect on *PERCC1* [37].

### The role of in silico prediction

We have applied four (SpliceAI, TraP-score, S-CAP-score, and CADD) [38–41] in silico prediction tools to all variants that have been empirically tested for their transcript-deleterious effect in this cohort (*n* = 169, including 4 from the literature) (Additional file 9: Table S7). To simplify the analysis, we used the default cutoff value suggested in each of these tools to classify variants as “deleterious” or “non-deleterious.” We found that none of these tools achieved > 71% sensitivity in predicting the pathogenic nature of the variants we tested at the RNA level (SpliceAI (65%), TraP-score (63%), S-CAP-score (61%), and CADD (71%) and that at least one of the four tools failed to predict the pathogenicity of 25% of the variants. However, the yield of these tools was widely different between the different classes. Only 8% of class (a) variants compared to 44.8% of the other classes combined (18% for class b, 46% for class c, 33% for class d, 10% for class e, neither of the two class f variants was empirically tested in this study) received inconsistent prediction in silico. In agreement with our suggestion that class (b) variants should be lumped with class (a) (for the purpose of assigning a canonical splicing score on the ACMG classification), we show that in no instance did the four tools disagree on classifying these variants as pathogenic.

### In search of tissue-specific aberrant transcripts

In addition to the 169 variants for which patient RNA material was available and tested, we also tested the expression of the genes containing the remaining 103 TDVs, in blood, skin, and urine (renal epithelial cells, see “Materials and methods”) derived RNA, since these are the readily available sources of RNA clinically. Please note that blood-derived RNA was extracted from PAXGene and/or LCL and these are listed separately in Additional file 2: Table S1. We found that 84.1% (195 out of 232) of the tested genes are expressed in the blood-derived RNA, 85.8% (199 out of 232) in fibroblast-derived RNA and 90% (209 out of 232) in the renal epithelial cells-derived RNA. The majority of genes were expressed in all three sources of RNA (75.5%), while only 2.6% (6 out of 232 genes) were not expressed in any of these sources. We were able to detect the aberrant transcript associated with TDVs in controls who lack the respective variant in only 11/169 (6.5%) of those that were empirically tested. In all these instances, the aberrant transcript was much less abundant in controls, and in none of these cases was the aberrant transcript listed in Ensembl or UCSC Genome Browser. In the 12 patients for whom we had both skin- and blood-derived RNA, we found no instance of an aberrant transcript that was solely present in one but not the other whenever the gene was expressed in both (*n* = 11, Additional file 2: Table S1). However, we did encounter two instances of pathogenic variants that did not reveal aberrant transcripts in blood-derived RNA (*FBN1*:NM_000138.4:c.6872-1G>A and *COL6A2*:NM_001849.3:c.1459-63G>A). We conclude that the deleterious effects of these variants may be tissue-specific.

## Discussion

RNA has long been exploited to investigate the effect of variants suspected to alter the final transcript. However, unbiased sequencing of all transcripts in an RNA sample (RNA-Seq) was only possible recently thanks to technological advancements. It is not surprising, therefore, that there is much enthusiasm about RNA-Seq as a supplemental test to genome sequencing to diagnose Mendelian conditions, among other indications. Although the effect of noncoding variants with GWAS significance on splicing is increasingly appreciated, the goal of this study was to study variants only in the context of Mendelian diseases since this is the area that stands to benefit most from the current applications of RNA-Seq [10, 19, 42–44]. Unlike the relatively homogeneous DNA, RNA is highly heterogeneous spatially and temporally. In addition, there is marked variability in the abundance of different transcripts even in a given cell. Finally, the effect of pathogenic variants on RNA is far more nuanced than the simple “present” or “absent” that characterizes DNA variants (even mosaic DNA variants are either present or absent in a given cell). These factors make the use of RNA-Seq in clinical diagnostics challenging and highlight the need for empirical data, e.g., mapping splicing variations in clinically accessible tissue, that inform the development of computational tools that unlock the full potential of this technology [45, 46].

This study is an attempt to contribute to the literature on RNA-based diagnosis of Mendelian diseases. The large volume of our cohort (2438 molecularly characterized Mendelian families) spanning 1807 Mendelian genes, and our unique resource of families that map to single loci and thus offer an unbiased window into the breakdown of disease-causing variants in Mendelian diseases, allowed us to draw several conclusions. First, we estimate the contribution of TDVs to be at least 15% of the overall Mendelian mutation pool, although our unbiased estimate based on single locus families suggests a higher contribution of 18.9%. This has important implications because it suggests that RNA-Seq has a great potential in solving Mendelian phenotypes. Unfortunately, it is not possible to compare this hypothetical yield to what has been achieved in the few reported studies since those studies heavily focused on cases that could not be diagnosed by WES or WGS [10, 11], including a recent study involving 94 individuals with undiagnosed rare diseases that suggested a diagnostic rate of 16.7% [19]. This yield is similar to our estimated yield (13.5%) based on extensive positional mapping and RNA analysis of 155 Mendelian cases that could not be diagnosed by WES. Second, our finding of aberrant transcripts not described in databases that are detected in controls, despite their very low frequency, recapitulates the challenge described in previous studies in identifying the signal from noise when interpreting RNA-Seq. We suggest that while greater in magnitude, this challenge is no different in principle from the challenge of identifying the candidate causal variant in WES/WGS, and that filters that improve the signal/noise ratio are even more acutely needed in RNA-Seq. For example, we show in this study that the use of autozygome coordinates drastically reduces the search space in RNA-Seq (up to a factor of 300 in one case). While we acknowledge this filter is not always applicable, it should be pursued even in the absence of clear history of consanguinity since its integration into existing WES/WGS is straightforward as has been shown before [3]. Third, despite the significant investment in the development of in silico prediction tools, these remain far from perfect and our data clearly show that at least 25% of transcript-deleterious variants would be missed by tested tools. This suggests that these tools cannot replace RNA-Seq, which will likely become a standard clinical test for cases with negative WES/WGS. Fourth, and reassuringly, our data also seem to alleviate concerns about access to the relevant tissue since only < 10% of the tested genes were not expressed at all in the three sources of RNA available to us. Whenever the gene was expressed, we were able to demonstrate the effect of splicing in at least one of the two sources of RNA, with only two instances where a clearly pathogenic splicing variant did not result in aberrant transcript in the only tissue available for the respective patient, i.e., blood, despite abundant expression. It should be emphasized here that the overwhelming majority of the tested variants involved brain pathologies in their phenotypic expression. Fifth, we show several instances of abnormal splicing with no resulting phenotype as well as normal splicing with resulting phenotype for well-established disease genes. The apparent non-penetrance in the former scenario could be alternatively explained by a tissue-specific effect, which could also explain the latter scenario. Fortunately, these appear to be the exception; however, they are useful reminders of the expected limitation of RNA-Seq on clinically accessible samples. Sixth, although our study did not specifically aim to compare splicing to other classes of variants, we think that the examples we encountered with respect to the phenotypic expression of homozygous vs compound heterozygous regulatory variants is noteworthy. This phenomenon, first described in the context of thrombocytopenia-absent radius (TAR) syndrome, has only rarely been invoked since, e.g., *SNORD118*-related cerebral microangiopathy leukoencephalopathy with calcifications and cysts [47], and *TXNL4A*-related Burn-McKeown syndrome [48]. We have previously shown that a non-canonical splice-site variant in *DONSON* causes microcephalic primordial dwarfism when inherited in trans with a hypomorphic variant, but results in an embryonically lethal microcephaly-micromelia syndrome when homozygous [49]. Here, we show that homozygosity for the most common disease-causing mutation in *SBDS* is not sufficient to cause SDS and that its inheritance in trans with a more severe mutation seems necessary. This calls for caution in inferring pathogenicity of a previously reported and confirmed pathogenic variants depending on their zygosity, and we suggest that regulatory and splicing variants may be particularly prone to this phenomenon.

In conclusion, we report the largest cohort of Mendelian phenotypes with comprehensive analysis of their underlying transcript-deleterious variants. The lessons learned from this cohort expand our knowledge of this class of variants and provide much needed empirical data for the clinical implementation of RNA-Seq as a promising supplemental tool to genome sequencing.

## Supplementary information

**Additional file 1.** Supplemental file 1. Causal gene prediction comparison for RNA-Seq data.

**Additional file 2.** Table S1. Full listing of the 272 transcript-deleterious variants identified in our cohort. * indicates genes that required more amplification cycles to detect expression.

**Additional file 3.** Table S2. Frequency of all transcript deleterious variants are reported in the current study.

**Additional file 4.** Table S3. List of families that map to a single locus and the outcome of their investigation.

**Additional file 5.** Table S4. Full list of negative clinical WES cases and the outcome of their analysis by RNA studies and other tools. ‘A’ indicates a novel gene/novel gene for the condition.

**Additional file 6. ** Figure S1. Pedigree of a family which is mapped to a single locus and identified a very deep (+ 335) variant in *ABCB4* causing cholestatic disease. Figure S2. A) A sashimi plot showing base-level densities of reads mapped to a genomic region containing exons 11 and 12 of *KCTD3* transcripts from three samples. The x-axis represents the genomic coordinate in hg19. The y-axis represents per-base read counts, and the range is specified in the upper-left corner of the plot for each sample. Arcs connecting exons represent splice junction reads. The horizontal bar lines on the bottom indicate isoforms (exons as rectangle boxes and introns as line with arrow heads). The distribution in blue shows the sample with aberrant *KCTD3* transcript, while the other two distributions are from randomly selected samples of lymphocytes (red from a patient and green from the GTEx cohort). B) A sashimi plot showing base-level densities of reads mapped to a genomic region in the positive control cases.

**Additional file 7.** Table S5. The performance comparison results of our RNA-Seq pipeline.

**Additional file 8.** Table S6. Transcript-deleterious variants with unusual phenotypic consequences.

**Additional file 9.** Table S7. In silico (using SpliceAI, TraP, S-CAP, and CADD) predictions for all transcript-deleterious variants in this cohort that were empirically tested.

**Additional file 10.** Review history.

## Data Availability

The raw RNA-seq data from 11 human samples are deposited in NCBI Sequence Read Archive under bioproject ID PRJNA625628 [50] (https://www.ncbi.nlm.nih.gov/bioproject/PRJNA625628). Source code is deposited in Zenodo [51]. Pipe line for Alpha/beta-based scoring and filtering of candidates is deposited in guthub [52]. Whole exome sequence data sets are deposited in NCBI under dbSNP [53]. The expression dataset of Genotype-Tissue Expression (GTEx) Common Fund Project is available through Genotypes and Phenotypes (dbGaP) under accession number phs000424.v8.p2 (third party data).
