# Supplementary material for: Analysis of transcript-deleterious variants in Mendelian disorders: implications for RNA-based diagnostics
Source: Genome Biol. 2020 Jun 17;21:145. doi: 10.1186/s13059-020-02053-9 (PMC7298854; doi:10.1186/s13059-020-02053-9)
Supplement: Supplementary file 1 — Additional file 1. Supplemental file 1. Causal gene prediction comparison for RNA-Seq data. [file 13059_2020_2053_MOESM1_ESM.pdf]

# Causal gene prediction comparison

## Settings

To evaluate the value of our transcriptome-based causal gene prediction pipeline, we compared its performance against that of six different approaches consisting of five splicing outlier detection approaches and one expression outlier prediction method. The splicing outlier detection approaches we used are: (1) the method used by Kremer et al. [8], which is based on leafcutter [9]; (2) another approach based on leafcutter with a stricter p-value cutoff; (3) SPOT which was used by Ferraro et al. [6]; (4) the approach used by Cummings et al. [4]; and (5) a different configuration of the Cummings et al.'s method which was used by Gonorazky et al. [7]. The expression outlier detection approach that we used for this comparison is OUTRIDER [2]. Because the purpose of this comparison is to evaluate the gene-level prediction accuracy, we called the prediction of a splicing outlier method correct if its prediction included any splicing outlier within the true causal gene. Thus, for example, while all of the splicing outlier detection methods used in this comparative analysis focus on only split reads, our evaluation allowed them to correctly predict a causal gene even when disease-specific aberrations are based on complete intron retention (such as the 15DG2154 case we have).

The datasets we used for this comparison are the six cases with known causal genes (the five known cases and the case in which KCTD3 was identified as the causal gene). We used the same set of RNA-seq datasets for the prediction. That is, 11 in-house datasets and 437 GTEx datasets [3] for the LCL (7 + 134) and the fibroblast (4 + 303) sample types. For each case, we used a set of coding genes located in the ROH as the potential candidates and evaluated the prediction performance by analyzing the correct prediction of the causal gene and the number of candidate genes. These results are shown in a supplementary table.

## The Kremer et al. splicing outlier prediction

To apply the splicing outlier prediction approach that Kremer et al. [4] devised, we first downloaded leafcutter [9]. We generated an annotated exon data file using GENCODE v25. We set parameter values for the scripts to be compatible with Kremer et al. To generate splicing junction clusters, we set minclureads to be 30 and maxintronlen to be 500,000. Because each case was compared against the rest of the in-house and GTEx datasets, the minimum number of samples in a group and for introns was set to 1 in the differential splicing analysis step. Splicing events with adjusted p-value $< 0.05$ were considered to be splicing outliers.

## Another leafcutter-based splicing outlier prediction

To evaluate splicing outlier prediction from leafcutter with a stricter cutoff value, we set 0.0027 as the adjusted p-value cutoff.

## SPOT

We downloaded SPOT from https://github.com/BennyStrobes/SPOT. We corrected the format of the cluster data output from leafcutter and used it as the input of SPOT. After running SPOT, we used 0.0027 as the p-value cutoff (i.e., $|\mathrm{z-score}| > 3$) as described by Ferraro et al. [6].

## The Cummings et al.'s splicing outlier prediction

To apply the Cummings et al.'s splicing outlier prediction method, we first downloaded the MendelianRNA-seq package at https://github.com/berylc/MendelianRNA-seq. We generated the gene list and annotated junction files using GENCODE v25. To make use of MendelianRNA with our aligned reads which were generated using STAR [5], we changed a piece of the code which intends to extract uniquely mapped split reads so that we could have the correct MAPQ value for uniquely mapped reads. Using the same filtering criteria specified by Cummings et al., a splicing junction in a given sample is considered to be a splicing outlier if the following conditions are satisfied: (*i*) its normalized junction score is at least 0.05; (*ii*) it is supported by at least 2 reads; (*iii*) its donor or acceptor end is annotated; (*iv*) the given sample has the highest value normalized junction score which is at least twice as high as the next highest normalized score.

## Gonorazky et al.'s configuration of the Cummings et al.'s splicing outlier prediction

To apply the splicing outlier prediction approach that Gonorazky et al. devised, we used the splicing outlier prediction method developed by Cumming et al. with the following filtering criteria. A splicing junction is considered as a splicing outlier if it satisfies all of the following five conditions: (*i*) it is present in at most 5 samples from GTEx control; (*ii*) it is not annotated in GENCODE v25; (*iii*) it has read support with at least 5 reads; (*iv*) it has a normalized junction score of at least 0.05; (*v*) it is not shared in more than 2 samples in our in-house patients' datasets.

## OUTRIDER

To apply OUTRIDER to obtain expression outliers, we first generated (unnormalized) gene-level counts for coding genes with Kallisto [1]. Since OUTRIDER depends on transformation based on a discrete probability distribution, we rounded the Kallisto estimates

to nearest one. Before running OUTRIDER, we used the expression filter function provided in the OUTRIDER package. We then ran the program with the default setting, which included 15 iterations of optimization.

# References

1. Nicolas L Bray, Harold Pimentel, Páll Melsted, and Lior Pachter. Near-optimal probabilistic RNA-seq quantification. *Nature biotechnology*, 34:525–527, May 2016.

2. Felix Brechtmann, Christian Mertes, Agnė Matusevičiūtė, Vicente A Yépez, Žiga Avsec, Maximilian Herzog, Daniel M Bader, Holger Prokisch, and Julien Gagneur. OUTRIDER: A statistical method for detecting aberrantly expressed genes in RNA sequencing data. *American journal of human genetics*, 103:907–917, December 2018.

3. GTEx Consortium. The Genotype-Tissue Expression (GTEx) project. *Nature genetics*, 45:580–585, June 2013.

4. Beryl B Cummings, Jamie L Marshall, Taru Tukiainen, Monkol Lek, Sandra Donkervoort, A Reghan Foley, Veronique Bolduc, Leigh B Waddell, Sarah A Sandaradura, Gina L O'Grady, Elicia Estrella, Hemakumar M Reddy, Fengmei Zhao, Ben Weisburd, Konrad J Karczewski, Anne H O'Donnell-Luria, Daniel Birnbaum, Anna Sarkozy, Ying Hu, Hernan Gonorazky, Kristl Claeys, Himanshu Joshi, Adam Bournazos, Emily C Oates, Roula Ghaoui, Mark R Davis, Nigel G Laing, Ana Topf, Genotype-Tissue Expression Consortium, Peter B Kang, Alan H Beggs, Kathryn N North, Volker Straub, James J Dowling, Francesco Muntoni, Nigel F Clarke, Sandra T Cooper, Carsten G Bönnemann, and Daniel G MacArthur. Improving genetic diagnosis in Mendelian disease with transcriptome sequencing. *Science translational medicine*, 9, April 2017.

5. Alexander Dobin, Carrie A Davis, Felix Schlesinger, Jorg Drenkow, Chris Zaleski, Sonali Jha, Philippe Batut, Mark Chaisson, and Thomas R Gingeras. STAR: ultrafast universal RNA-seq aligner. *Bioinformatics (Oxford, England)*, 29:15–21, January 2013.

6. Nicole M. Ferraro, Benjamin J. Strober, Jonah Einson, Xin Li, Francois Aguet, Alvaro N. Barbeira, Stephane E. Castel, Joe R. Davis, Austin T. Hilliard, Bence Kotis, YoSon Park, Alexandra J. Scott, Craig Smail, Emily K. Tsang, Kristin G. Ardlie, Themistocles L. Assimes, Ira Hall, Hae Kyung Im, , Tuuli Lappalainen, Pejman Mohammadi, Stephen B. Montgomery, and Alexis Battle. Diverse transcriptomic signatures across human tissues identify functional rare genetic variation. *bioRxiv*, 2019.

7. Hernan D Gonorazky, Sergey Naumenko, Arun K Ramani, Viswateja Nelakuditi, Pouria Mashouri, Peiqui Wang, Dennis Kao, Krish Ohri, Senthuri Viththiyapaskaran, Mark A Tarnopolsky, Katherine D Mathews, Steven A Moore, Andres N Osorio, David Villanova, Dwi U Kemaladewi, Ronald D Cohn, Michael Brudno, and James J Dowling. Expanding the boundaries of RNA sequencing as a diagnostic tool for rare mendelian disease. *American journal of human genetics*, 104:466–483, March 2019.

8. Laura S Kremer, Daniel M Bader, Christian Mertes, Robert Kopajtich, Garwin Pichler, Arcangela Iuso, Tobias B Haack, Elisabeth Graf, Thomas Schwarzmayr, Caterina Terrile, Eliška Koňaříková, Birgit Repp, Gabi Kastenmüller, Jerzy Adamski, Peter Lichtner, Christoph Leonhardt, Benoit Funalot, Alice Donati, Valeria Tiranti, Anne Lombes, Claude Jardel, Dieter Gläser, Robert W Taylor, Daniele Ghezzi, Johannes A Mayr, Agnes Rötig, Peter Freisinger, Felix Distelmaier, Tim M Strom, Thomas Meitinger, Julien Gagneur, and Holger Prokisch. Genetic diagnosis of Mendelian disorders via RNA sequencing. *Nature communications*, 8:15824, June 2017.

9. Yang I Li, David A Knowles, Jack Humphrey, Alvaro N Barbeira, Scott P Dickinson, Hae Kyung Im, and Jonathan K Pritchard. Annotation-free quantification of RNA splicing using LeafCutter. *Nature genetics*, 50:151–158, January 2018.
