# Supplementary material for: Analysis of transcript-deleterious variants in Mendelian disorders: implications for RNA-based diagnostics
Source: Genome Biol. 2020 Jun 17;21:145. doi: 10.1186/s13059-020-02053-9 (PMC7298854; doi:10.1186/s13059-020-02053-9)
Supplement: Supplementary file 6 — Additional file 6. Figure S1. Pedigree of a family which is mapped to a single locus and identified a very deep (+ 335) variant in ABCB4 causing cholestatic disease. Figure S2. A) A sashimi plot showing base-level densities of reads mapped to a genomic region containing exons 11 and 12 of KCTD3 transcripts from three samples. The x-axis represents the genomic coordinate in hg19. The y-axis represents per-base read counts, and the range is specified in the upper-left corner of the plot for each sample. Arcs connecting exons represent splice junction reads. The horizontal bar lines on the bottom indicate isoforms (exons as rectangle boxes and introns as line with arrow heads). The distribution in blue shows the sample with aberrant KCTD3 transcript, while the other two distributions are from randomly selected samples of lymphocytes (red from a patient and green from the GTEx cohort). B) A sashimi plot showing base-level densities of reads mapped to a genomic region in the positive control cases. [file 13059_2020_2053_MOESM6_ESM.pdf]

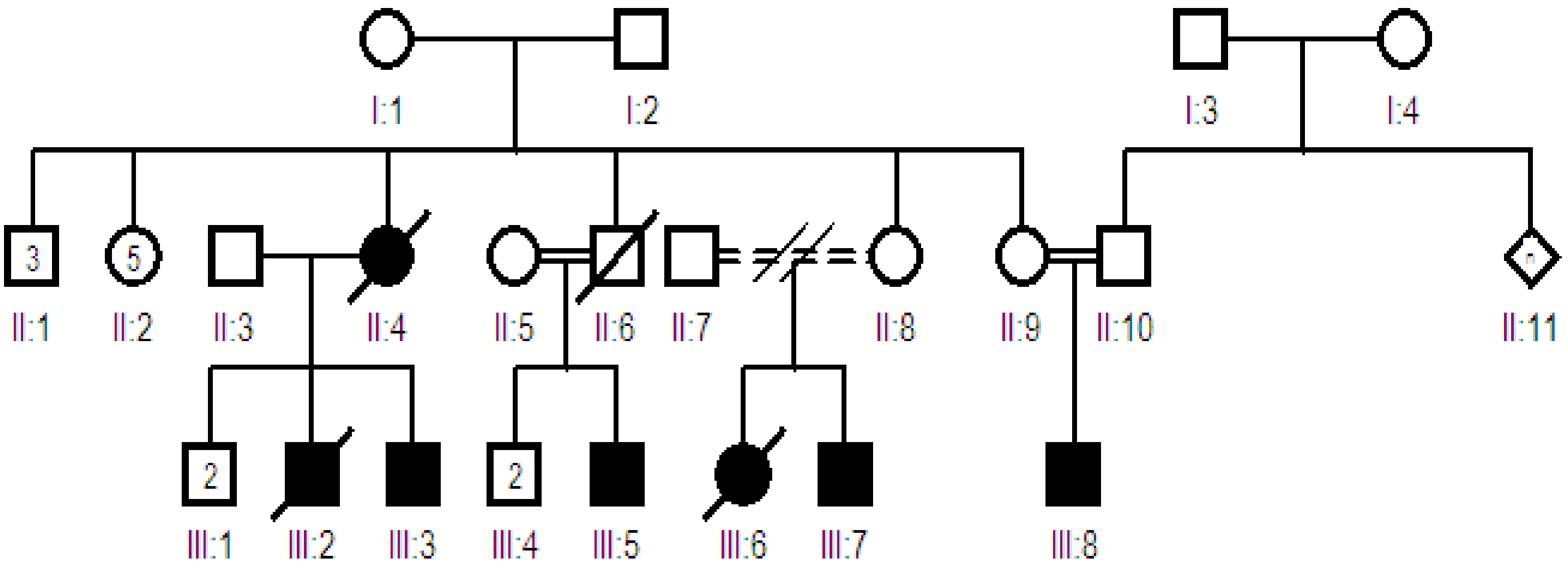

ABCB4 NC_000007.13:g.87091739T>C NM_000443.3:c.286+335A>G

Figure S1. Pedigree of a family which is mapped to a single locus and identified a very deep (+335) variant in *ABCB4* causing cholestatic disease.

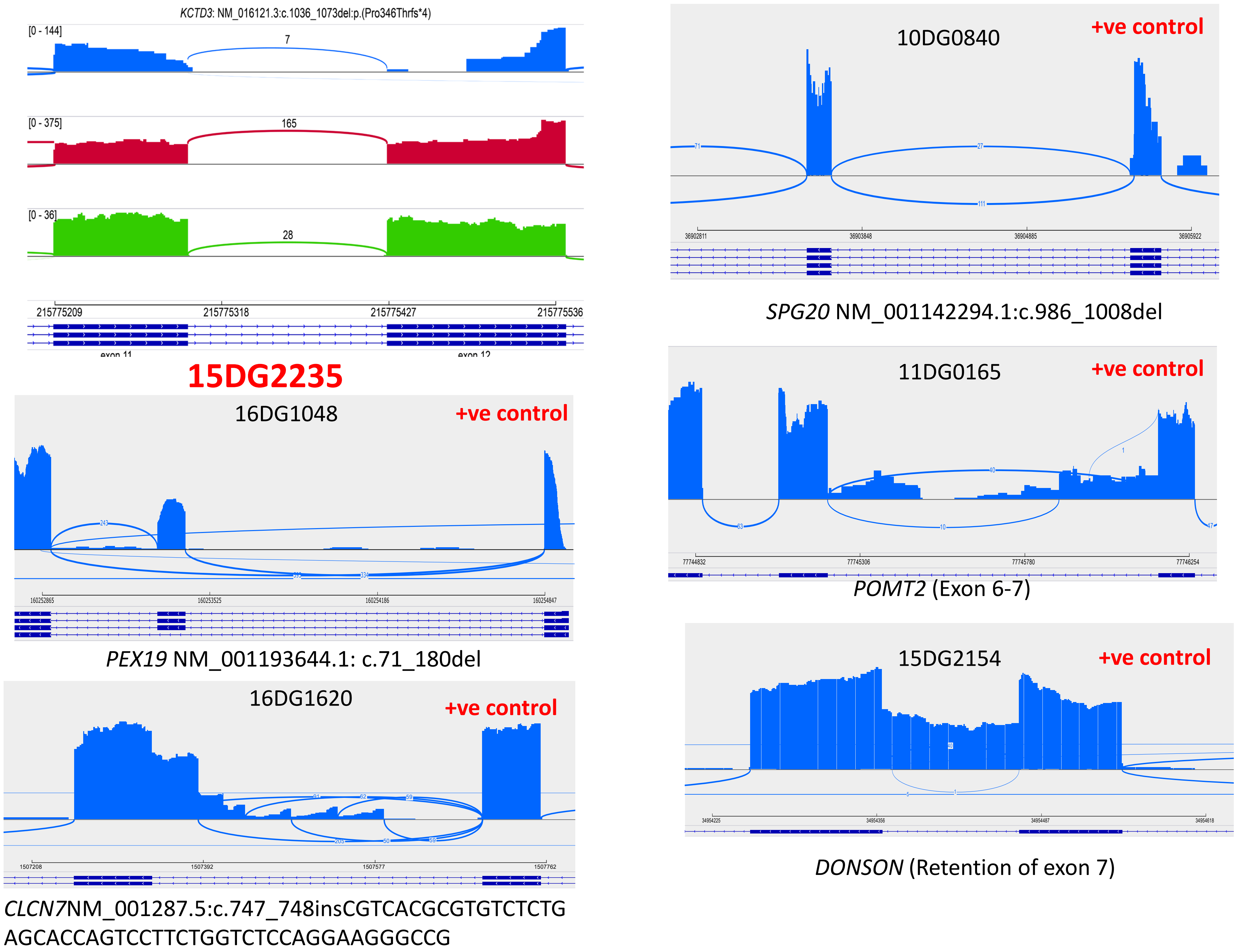

Figure S2. A) A sashimi plot showing base-level densities of reads mapped to a genomic region containing exons 11 and 12 of *KCTD3* transcripts from three samples. The x-axis represents the genomic coordinate in hg19. The y-axis represents per-base read counts, and the range is specified in the upper-left corner of the plot for each sample. Arcs connecting exons represent splice junction reads. The horizontal bar lines on the bottom indicate isoforms (exons as rectangle boxes and introns as line with arrow heads). The distribution in blue shows the sample with aberrant *KCTD3* transcript, while the other two distributions are from randomly selected samples of lymphocytes (red from a patient and green from the GTEx cohort). B) A sashimi plot showing base-level densities of reads mapped to a genomic region in the positive control cases.
