# Supplementary material for: Analysis of transcript-deleterious variants in Mendelian disorders: implications for RNA-based diagnostics
Source: Genome Biol. 2020 Jun 17;21:145. doi: 10.1186/s13059-020-02053-9 (PMC7298854; doi:10.1186/s13059-020-02053-9)
Supplement: Supplementary file 10 — Additional file 10. Review history. [file 13059_2020_2053_MOESM10_ESM.docx]

Review History

**First round of review**

**Reviewer 1**

**Are you able to assess all statistics in the manuscript, including the appropriateness of statistical tests used?** There are no statistics in the manuscript.

**Comments to author:**

This is a paper that reports two aspects of findings related to disruption of mRNA of rare inherited DNA variants.  The first component is the analysis of DNA variants that the authors deem to be likely causal of disease and could be interpreted from exome data. This represents 14% of all solved cases (n=255 variants).  The other 86% of their solved cases are from known or strongly predicted to be pathogenic DNA variants within the coding sequencing, but presumably NOT disrupting splicing.  The authors break the types of DNA mutations into a series of categories for their effect on splicing/expression. Not all of the variants are confirmed, but a large portion are.  Many of the mutations are highly likely to predict a consequence to mRNA and thus to protein, and are thus less interesting, and also falsely inflating the novelty of their work.   The authors call all of these 'Mendelian' families.  I suspect that this means that there is a family history and rare phenotype consistent with a known or unknown genetic disease.  The exact criteria of declaring a case 'solved' is not specified.  The 'Mendelian' aspect may impact the generality of their findings and their approach, but nonetheless the authors do add to the important literature regarding the necessity of using RNA analysis, especially RNASEQ to query the genome more broadly and appropriately interpret intronic, exonic, UTR portions of the genome, which are currently inadequately interpreted, even if DNA variants identified.

The basis of 'mapping to single loci' is not clear and should be clarified in the manuscript.  When the authors take the subset of cases that appear to have a single interval in the genome that could be causal and consistent with autosomal recessive inheritance (this is a question), then the percentage of all of the cases that are 'solved' rises from 14% in the overall cohort to 18%. I am not at all clear why the authors call this an 'unbiased' sampling. This logic does not make sense to me and needs to be clarified in the manuscript.  Perhaps the issue is that the authors are NOT really looking genome-wide and are not considering all possible splice altering DNA mutations or other mutations that might alter RNA? And thus because the breadth of potential DNA variants that could be causal, given other familial information and linkage, that the RNA data could be inspected more deeply or a larger number of potentially causal variants could be considered in a much small list of candidate genes? Regardless, this needs substantial revision to clarify the thought process of the authors and the meaning intended.

The value of the first phase of this work is limited in scope and perhaps not stated correctly, given the known importance of the last base of each exon and the first two bases into the intron, these positions are often deemed 'likely pathogenic'… what is not clear from that interpretation, common in clinical laboratories, is the actual affect on mRNA. A canonical splice site mutation may extend the exon, cause exon skipping, or even have broader consequences on splicing.  Thus an important point is that, when possible, anaylsis of RNA is beneficial in these instances to determine if an inframe mutation is made, or a frameshifting mutation is made, and in frame mutation that introduces a stop codon, etc.  

IT is obvious that many splice altering alleles are not observed in current exome data, table S1 only has two genes in the version that I could download, so I cannot comment broadly on what most of the variants were that were detected.

In Table S2, there are some issues….

13DG1590      APC      NM_000038.5:c.423-4_423-2delATA:r.423_531del     Homozygous     Transcript deleterious variant (c)

Is the above appropriate categorized as 'c' as it would seem to be disrupting the canonical splice site?

The variants that are in class a or b and relatively obvious. However, their analysis of the total 11 last base position of an exon causing mRNA disruption highlights the importance of this base in each exon and if mutated is likely to disrupt the mRNA and protein.

The category c is the most interesting and 'non-obvious'. Since introns are very large relative to exons or the distance that their data cn look into the introns, this dataset and mutation type is of course likely to harbor many of the mutations that need to be discovered to alter mRNA. In total there were 9 or 156 of the mutations in Table S2 that were deeper intronic variants. This seems like a higher amount than others have found, and may be due to a willingness to consider homozygous intronic variants within a region of linkage by follow up with RT-PCR. IT would be good for the authors to analyse others data to determine if this is unusual in their cohort. Their cohort is uniquely powerful as the vast majority of affecteds have autosomal recessive diseases due to a homozygosity.

In the subset of the sample that was 'negative' by exome, they authors used RNA sequencing of the entire transcriptome from blood or fibroblasts, with some confirmations by RTPCR, of the disruption of mRNA due to rare DNA mutations.  The work is somewhat confusing to read and in its presentation, but important points are contributed by this manuscript to the literature.  Overall the introduction should be shortened, and the authors should remove reduncancies such as

"However, the anticipated attainment of the maximum diagnostic yield through whole-genome sequencing (WGS) has not been realized. The minimal boost of diagnostic yield offered by WGS over WES suggests that the bottleneck is not in the capture/calling of the causal variants in the sequencing stage but rather in their interpretation [4, 5]. This notion is supported by studies showing the value of careful reinterpretation of "negative" WES and how misinterpreting the causal variants in WES is a major challenge that cannot be circumvented by WGS [5, 6]."  

These three sentences say the same thing and make the manuscript much more difficult to read, and examples can be found throughout the introduction, results and discussion.

The references for the manuscript are not entirely appropriate.  For instance, references 1-2 that are cited are not as relevant as the two below which make the same points but are published much earlier.

Clinical whole-exome sequencing for the diagnosis of mendelian disorders.
Yang Y, Muzny DM, Reid JG, Bainbridge MN, Willis A, et al.
N Engl J Med. 2013 Oct 17;369(16):1502-11. doi: 10.1056/NEJMoa1306555. Epub 2013 Oct 2.

Clinical exome sequencing for genetic identification of rare Mendelian disorders. Lee H, Deignan JL, Dorrani N, Strom SP, Kantarci S, et al.
JAMA. 2014 Nov 12;312(18):1880-7. doi: 10.1001/jama.2014.14604.


I would also add in the following references directly relevant and the authors should consider reasons for their high observation rate of disease causing DNA mutations that affect splicing relative to the prior work.

Lee H, Huang AY, Wang LK, Yoon AJ, Renteria G, et al. Diagnostic utility
of transcriptome sequencing for rare Mendelian diseases. Genet Med. 2019 Oct 14.
doi: 10.1038/s41436-019-0672-1. [Epub ahead of print] PubMed PMID: 31607746.


Finally, the authors should remove genes and data from table S3 that include the proposed 'novel genes' that will be reported elsewhere, as it is not possible to evaluate in this manuscript in this manner.

**Reviewer 2**

**Are you able to assess all statistics in the manuscript, including the appropriateness of statistical tests used?** Yes, and I have assessed the statistics in my report.

**Comments to author:**

Maddirevula and colleagues reported on the utility of RNA-based assays for diagnosis of Mendelian disorders using their large cohort of 5647 families with suspected Mendelian diseases. The study provides additional data into the diagnostic yield of RNA data complementing several studies that now exist in the literature. The value of their investigation has several interesting observations including an estimate that 14-18% of recessive Mendelian disorders can be mapped to transcript-deleterious variants and that tissue-specificity of transcript-deleterious effects is likely not too important. Overall, I found the manuscript interesting but have the following comments:

1)     The abstract is long and overly focuses on the large cohort. However, the cohort is not used for the majority of the novel features of the paper beyond describing the distribution of transcript-deleterious variants. The authors should focus on shortening the abstract and making clear the most important points.

2)     Estimation of likely causal variants driven by structural variants is underestimated and not calculated. Many of these SVs may have transcript deleterious impacts and this may increase the percentages beyond 18%. Many of the 43% without a causal variant from the 154 select cases could be transcript-deleterious and driven by more complex variant types.

3)     The alpha and beta scores seem largely uniformative for recovering candidate genes. The most impactful filter is the autozygome coordinate-filtering. The authors should comment more on this and how to successfully interpret these scores.

4)     How do the alpha/beta scores compare to much simpler expression and splicing Z-scores?

5)     A method called Outrider, AJHG, has been published for normalization of RNA-seq and detection of rare disease genes. Have the authors evaluated the use of this approach on their data?

6)     The abstract highlights a discussion of penetrance but this is not clearly provided in the results. Instead there are results about canonical splicing variants in disease genes with no phenotype and potential tissue-specificity of some of these effects. This should be removed from the abstract or discussed more clearly.

7)     Many of the supplemental table legends were cut-off in the PDF. Please provide an index of tables and their legends as a separate page at the end of the manuscript.

8)     The observations that the majority of transcript deleterious variants could be found between tissues is encouraging and an important observation. Is there more overlap of studied disease genes between tissues than expected by chance? Could this percentage be calculated directly from OMIM or other data?

9)     What allele-frequency filters were used, if any? Can the authors report the allele frequency distribution of their different classes of variants?

10)     Does existing phenotype information aid in recovering target genes? Approaches like AMELIE have been designed to help reduce the scope of target genes further. How would these data converge in their positional filtering? My concern is whether RNA-seq here gives anything more than this other type of approach.

11)     The paper focuses mostly on homozygous recessive alleles and would benefit from making this clearer. There are approaches using RNA-seq such as ASE-based analyses that are not possible to leverage here but that may yield more traction in other areas. Please see the recent paper by Mohammadi in Science, 2019 for ANEVA.

12)   The authors need a more specific title to clarify their works impact. The title is too general particularly with existing studies in this area.

**Reviewer 3**

**Are you able to assess all statistics in the manuscript, including the appropriateness of statistical tests used?** Yes, and I have assessed the statistics in my report.

**Comments to author:**

Maddirevula et al retrospectively analyze the contribution of variants affecting transcripts in a large cohort (5,647 families) of patients with a suspected Mendelian disorder. To move beyond coding variant analysis, they introduce the notion of "transcript deleterious" variants and perform a series of experimental validation transcriptome assays to asses their role. Overall, the manuscript is hard to follow, lacks a clear message, is confusing and misleading. It generally lacks systematic assessments, justifications of the claims, and comparisons to the state-of-the-art. The two main figures are descriptive and lack a conclusive message. I suggest rejection. I provide below major and minor comments which hopefully will help the authors for future analyses of the data.


Major comments
----------

It is hard to understand what the key point of the paper is. The abstract is extremely long without a clear structure delineating the addressed question, main claims, and implication.


The title "RNA-based diagnosis of Mendelian disorders" suggests that the paper will discuss cases that were solved with the aid of RNA-Seq. However, RNA-Seq was performed on 6 exome negative cases, out of which they were able to solve 1. The manuscript is instead articulated around assessing the association of their proposed "transcript-deleterious variants" with disease, and to some extent, with aberrant expression and aberrant splice isoform usage of the respective genes. However, the concept of "transcript deleterious" variants is unclear.

The concept or transcript-deleterious variant, which is central to the paper, is unclear. The abstract defines those variants as "variants likely to cause their phenotypes by interfering with the normal transcription of the respective gene". This is wrong. Transcription refers to RNA synthesis. In contrast, the variants considered by the authors include variants affecting RNA processing (in particular splicing). Literally, a "transcript-deleterious variant" should refer to any variant deleterious for the transcript. However, they excluded protein-truncating coding variants (according to the abstract) although these variants potentially affect RNA stability by triggering non-sense mediated decay. Moreover, the variants are defined based on in silico criteria. Should not they be therefore designated as "potentially deleterious"? An alternative definition could be "transcript-encoded variants" but would then variants located outside the processed RNA such as intronic variants be considered? Without a clear conceptual definition of this central notion, the study falls apart.

The executive definition of the transcript-deleterious variants is also unclear. In particular, what are "noncanonical splice site intronic variants" (Class c)? Also, which algorithm has been used to identify "promoter/enhancer elements" (Class f)?

Variants were classified as candidate transcript-deleterious if they were compatible with pathogenicity potential in terms of frequency and segregation. However, there are no details of the cutoffs used.

The authors apply 4 different prediction tools to the 6 classes of variants that they define. They find that 7% of "class a" variants have inconsistent predictions among the 4 methods, but from the other 5 classes combined, as much as 44.8% of the variants have inconsistent predictions. Aggregating the results of the 5 groups is misleading. Moreover, it is unclear what the goal was when applying the methods. Splice-AI, T-RAP, S-CAP are splicing predictors whereas CADD is a general-purpose score. This should be explained and taken into account in the interpretation of the results.

It is very difficult to follow the sample number breakdown (the main text is as confusing as the abstract). An overview diagram of the workflow showing the number of samples entering each stage (WES, RNA-seq, RT-PCR), the numbers of solved and unsolved, the numbers of affected and unaffected, would be helpful. Also, the authors should make a clear distinction between the number of families, the number of individuals, and the number of affected individuals.


For a better understanding of the data, more details should be provided about the 5,647 families with suspected Mendelian phenotypes. Are these trios? Are these consanguineous families? Is there WES for all? How many individuals? What fraction of these families show autosomal recessive mode of inheritance?

Based on what criteria "a likely causal variant was identified in 2,429 of these families"? How is the functional relationship between genotype and phenotype assessed? In fact, there are only 156 families where Homozygosity track leads to a single locus, it is thus unclear how "likely causal" the variants of these 2,429 families are.

Analysis of RNA-seq data is based on ref. 26, which is a non-peer-reviewed preprint of some of the authors. This preprint is poorly written. It is supposed to increase the sensitivity to detect genes with an aberrant splicing pattern but no assessment for that claim is provided. The only benchmark is a clustering of GTEx samples which is claimed to be more accurate than based on TPM. Clustering is not a piece of evidence for improved detection of aberrant splicing events. No ROC-curve nor precision-recall against splicing outlier detection methods (pipelines described by Cummings et al. /Gonorazky et al.,  Kremer et al, and Fresard et al.) is given. How is the overlap? Is there evidence for higher enrichment of rare variants or disease-causing variants. It would be also interesting to compare it to SPOT (Ferraro et al, bioRxiv, https://www.biorxiv.org/content/10.1101/786053v1, online prior to the submission of this paper). Moreover, RNA-seq can lead to aberrant levels, aberrant splicing and mono-allelic expression (Kremer et al. Nat. communs, 2017). Only aberrant splicing is assessed with Omega as far as I understand. This should be clarified.

The second criterion for a gene to be considered as an RNA candidate is that it must have the lowest alpha_g value out of all the other patients. Therefore, each gene can be an outlier of at most one patient, which even in rare diseases is not true. It would be good to know how many candidates were found in total.

The abstract states that the RNA analysis could establish a diagnosis in ~13% of 155 patients with exome negative results. However, the main text refers to 154 patients out of which 57% were solved after re-analysis of WES and 18% of which harboured a transcript-deleterious variant. The ~13% of the abstract is never mentioned in the text. On the contrary, from the text (and Fig 2B which is a pie chart with ⅙ colored), it seems that RNA-Seq helped solving 1 sample (Fig 2C)  out of 6 tested.

There is no data or quantification provided for the RT-PCR measurements. A figure providing the number of samples and results would be helpful. The abstract states that definitive results from RT-PCR could be obtained for 83.4% variants, but this is not shown in Results.


The discussion states that the autozygome coordinates filter reduces the search space by a factor of 300. However, this number is never mentioned throughout the manuscript. Moreover, expression or splicing outliers detection followed by false discovery rate cutoffs give a handful of candidates genes on RNA-seq and thus this filter might not be needed with state-of-the-art methods (see Kremer et al, Nat communs. 2017; Brechtmann et al. AJHG 2018 -- there is also a recent preprint Mertes et al. biorxiv 2019).

For reproducibility, the source code should be shared. Most crucial is the source code to classify variants in either of the categories a-f.


Bottom of page 13: "A likely causal variant was subsequently identified in 57%." What does this mean? What are the criteria applied? Howe many variants these criteria yield per sample?

It is unclear how Table 1 is established and why these represent solved cases. I picked up one example. DCDC2 is listed for patients with bile diseases (cholastic) but DCDC2 is not expressed in liver and has been so far associated with developmental dyslexia, which does not appear to be related to bile. This is just one example. For every gene, the relationship between presented phenotypes and gene function should be explained otherwise the causality seems dubious.

P. 14 "These include a very deep (+335) variant in ABCB4 causing cholestatic disease in several affected members of an extended family" Why not with all affected members? Pedigrees must be provided to assess the reliability of the disease-association claims.


P.19 "Our results show that only 9.5% of these genes were not expressed in either RNA source." This claim should be substantiated with the number of genes reported per tissue, Venn diagram, and Method details provided. It feels rather low but it is all about a threshold issue. This statement is representative of the entire manuscript where figures are provided without point of reference, ways to assess the reliability of the number, details in the procedure, and comparison with the literature. Also, this falls short. Investigation of GTEx tissues could corroborate further this hypothesis. Have these genes or exons indeed some tissue-specificity? If so, is the tissue / the affected tissue or related to the phenotype?

Minor comments
---------

Abstract and p. 7: the term "premature truncating coding variants" does not exist. It is either "protein-truncating variant" or "premature termination codon".


p. 8 "invariant splicing codes": this term is unclear and does not exist in the literature.

The 4 tested prediction tools are not cited.


Gonorazky et al. AJHG 2019 should be referred to and compared to.

Aicher et al, biorxiv https://www.biorxiv.org/content/10.1101/727586v1 should be discussed.

P. 13 "Table S3 shows that many of these cases harbored a likely deleterious variant in a gene that was novel at the time". What does "the gene was novel" mean? Not known? Not annotated? Not associated with any disease? Not associated with this disease?

P. 18 "Class b should be lumped with class a": there is probably a selection bias if the variants were selected to associate with the disease. Actually, effects of splice dinucleotides variants (which define splice sites) on splicing are stronger than effects of variants on the two flanking nucleotides (which have a strong quantitative effect but are not splice site defining).


In general, the Materials and Methods section lack content.

**Authors Response**

**Point-by-point responses to the reviewers’ comments:**

Reviewer #1: We sincerely thank the respected reviewer for a very thorough and extremely insightful and helpful review. Below, please find our response:

1.The authors break the types of DNA mutations into a series of categories for their effect on splicing/expression. Not all of the variants are confirmed, but a large portion are.

*As the respected reviewer will appreciate, RNA material is not always available from patients for a variety of reasons. We did attempt to empirically test the effect of each of the mutation categories whenever an RNA source was available. While the paper was reviewed, we have managed to obtain RNA material to test additional 23 variants, which are now added. Please note that we specifically refrained from using mini-gene assays and relied solely on actual patient material because we wanted our data to be as closely reflective of actual biology as possible, including the observation that certain genes cannot be studied in blood derived cell lines.*

2- Many of the mutations are highly likely to predict a consequence to mRNA and thus to protein, and are thus less interesting, and also falsely inflating the novelty of their work.

*We would like to take this opportunity to clarify that we had to test the consequences of all categories empirically and not just assume that certain categories are highly likely to predict a consequence to mRNA. We believe this consistency was essential to make an unbiased determination of the sensitivity of the existing in silico tools in predicting a splicing effect. Additionally, many may be surprised that variants with an apparently obvious impact on splicing like the first and last base-pairs of an exon are not listed as such by the most authoritative guidelines on variant classification to date i.e. ACMG Guidelines. These guidelines list the various classes of PVS1 (loss of function) including canonical +1/2 bp position of introns but do not include the first and last bp of exons. We hope our work that consistently shows the deleterious impact of these variants on splicing will inform future updates of these guidelines.*

3- The authors call all of these 'Mendelian' families. I suspect that this means that there is a family history and rare phenotype consistent with a known or unknown genetic disease. The exact criteria of declaring a case 'solved' is not specified. The 'Mendelian' aspect may impact the generality of their findings and their approach, but nonetheless the authors do add to the important literature regarding the necessity of using RNA analysis, especially RNASEQ to query the genome more broadly and appropriately interpret intronic, exonic, UTR portions of the genome, which are currently inadequately interpreted, even if DNA variants identified.

*We regret not making ourselves clear in defining the term, which we now do in the revised manuscript as follows:*

*Original “Subjects described in this study represent combined cohorts recruited under individual IRB-approved research protocols (KFSHRC RAC# 2121053, 2080006 and 2070023). Informed consent was obtained from all subjects prior to their enrollment.” Revised “Subjects described in this study represent combined cohorts recruited under individual IRB-approved research protocols (KFSHRC RAC# 2121053, 2080006 and 2070023). In each of these protocols, we selectively recruited individuals with at least one of the following features a) positive family history consistent with a Mendelian inheritance of the disorder, and b) phenotypic presentation consistent with a previously published Mendelian disease. Informed consent was obtained from all subjects prior to their enrollment.”*

*As the respected reviewer can appreciate, we had to limit this study to the role of transcript-deleterious variants in the context of Mendelian disorders because this is the application that is most poised for clinical application and the one we can address with our dataset. In response to the respected reviewer’s suggestion, we have now added the following new sentence:*

*“Although the effect of noncoding variants with GWAS significance on splicing is increasingly appreciated, the goal of this study was to study variants only in the context of Mendelian diseases since this is the area that stands to benefit most from the current applications of RNA-Seq [Rentas, S., Rathi, K.S., Kaur, M. et al. Diagnosing Cornelia de Lange syndrome and related neurodevelopmental disorders using RNA sequencing. Genet Med (2020) doi:10.1038/s41436-019-0741-5, Fresard L, Smail C, Ferraro NM, et al. Identification of rare-disease genes using blood transcriptome sequencing and large control cohorts. Nat Med. 2019;25:911–919., Gonorazky HD, Naumenko S, Ramani AK, et al. Expanding the boundaries of RNA sequencing as a diagnostic tool for rare Mendelian disease. Am J Hum Genet. 2019;104:466–483., Cummings BB, Marshall JL, Tukiainen T, et al. Improving genetic diagnosis in Mendelian disease with transcriptome sequencing. Sci Transl Med. 2017;9:eaal5209., Kremer LS, Bader DM, Mertes C, et al. Genetic diagnosis of Mendelian disorders via RNA sequencing. Nat Commun. 2017;8:15824.]”.*

4- The basis of 'mapping to single loci' is not clear and should be clarified in the manuscript. When the authors take the subset of cases that appear to have a single interval in the genome that could be causal and consistent with autosomal recessive inheritance (this is a question), then the percentage of all of the cases that are 'solved' rises from 14% in the overall cohort to 18%. I am not at all clear why the authors call this an 'unbiased' sampling. This logic does not make sense to me and needs to be clarified in the manuscript. Perhaps the issue is that the authors are NOT really looking genome-wide and are not considering all possible splice altering DNA mutations or other mutations that might alter RNA? And thus because the breadth of potential DNA variants that could be causal, given other familial information and linkage, that the RNA data could be inspected more deeply or a larger number of potentially causal variants could be considered in a much small list of candidate genes? Regardless, this needs substantial revision to clarify the thought process of the authors and the meaning intended.

*This is an extremely helpful criticism because this is such an important part of the manuscript that our failure to communicate properly will greatly limit the manuscript’s impact and reach. Please allow us to explain the concept as follows. There is a growing concern that WES (whole-exome sequencing) may have reached its diagnostic limit and that WGS (whole-genome sequencing) should be implemented instead to detect non-coding variants that are presumed to underlie Mendelian cases that remain negative after WES. However, the question of whether most WESnegative cases are indeed caused by non-coding variants that are not captured by WES must ideally be addressed empirically by answering another question: what is the proportion of Mendelian diseases-causing* *variants that reside within the uncaptured part of the genome during WES? This is a very difficult question to address because the denominator and enumerator are under a very strong negative bias. Indeed, investigators are much more likely to identify variants in the coding and flanking noncoding part of the genome than in deeper parts of the genome. In order to circumvent this bias, we propose to resort to the “unbiased” nature of positional mapping where a phenotype is mapped to a single locus regardless of the nature of the causal variant. In other words, diseases caused by easily identifiable mutations and those caused by challenging deep intronic mutations are just as amenable to positional mapping. So if we focus on families in which Mendelian phenotypes can be mapped to single loci (this is typically possible when we have sufficient numbers of affected and unaffected members in a given family), we can have an “unbiased” breakdown of the classes of disease-causing variants in humans. This is indeed what we achieved in this study by analyzing 157 families in which we had been able to map various Mendelian phenotypes to single loci, the largest collection of single locus mapped phenotypes in any study to date to the best of our knowledge.*

5- The value of the first phase of this work is limited in scope and perhaps not stated correctly, given the known importance of the last base of each exon and the first two bases into the intron, these positions are often deemed 'likely pathogenic'… what is not clear from that interpretation, common in clinical laboratories, is the actual affect on mRNA. A canonical splice site mutation may extend the exon, cause exon skipping, or even have broader consequences on splicing. Thus an important point is that, when possible, anaylsis of RNA is beneficial in these instances to determine if an inframe mutation is made, or a frameshifting mutation is made, and in frame mutation that introduces a stop codon, etc

*This is another opportunity for us to highlight that what may seem obvious to many is actually not that obvious even in the most widely used guidelines. As we explained above, the first and last bp of exons are actually currently not listed as PVS1 by the ACMG guidelines. Thus, we hope that our data will help change that in future iterations of the guidelines.*

6- IT is obvious that many splice altering alleles are not observed in current exome data, table S1 only has two genes in the version that I could download, so I cannot comment broadly on what most of the variants were that were detected.

*We are a bit unclear about this comment by the respected reviewer. Table S1 lists all 273 variants that belong to the various categories of transcript-deleterious mutations and the effect of these variants on splicing as empirically tested using patient material. Perhaps this comment is prompted by the fact that Table S1 upon rendering to PDF was truncated, but we now asked the editorial office to provide this and all other tables as Excel files to this and the other respected reviewers.*

7- In Table S2, there are some issues…. 13DG1590 APC NM_000038.5:c.423-4_423-2delATA:r.423_531del Homozygous Transcript deleterious variant (c) Is the above appropriate categorized as 'c' as it would seem to be disrupting the canonical splice site?

*The respected reviewer is absolutely correct in that this deletion by extending to the canonical -2 position should have been classified as category “a”, which is how we classified it in the revised submission.*

8- The variants that are in class a or b and relatively obvious. However, their analysis of the total 11 last base position of an exon causing mRNA disruption highlights the importance of this base in each exon and if mutated is likely to disrupt the mRNA and protein.

*Thank you very much for the encouraging feedback!*

9- The category c is the most interesting and 'non-obvious'. Since introns are very large relative to exons or the distance that their data cn look into the introns, this dataset and mutation type is of course likely to harbor many of the mutations that need to be discovered to alter mRNA. In total there were 9 or 156 of the mutations in Table S2 that were deeper intronic variants. This seems like a higher amount than others have found, and may be due to a willingness to consider homozygous intronic variants within a region of linkage by follow up with RT-PCR. IT would be good for the authors to analyse others data to determine if this is unusual in their cohort. Their cohort is uniquely powerful as the vast majority of affecteds have autosomal recessive diseases due to a homozygosity.

*The respected reviewer is absolutely correct in their conclusion. It is precisely because of the advantage of “knowing where to look” thanks to positional mapping that we were able to identify almost all of the most challenging deep intronic variants. We do think these variants are underrepresented in other cohorts where this advantage is lacking so a direct comparison will be challenging and potentially misleading even. However, the respected reviewer will be pleased to know that in two previous studies, the largest of their kind on ciliopathies, we have indeed shown a higher percentage of noncanonical deep intronic variants that were able to identify thanks to homozygosity analysis (see PMID: 27894351 and Shamseldin et al (Genetics in Medicine, in press)).*

10- In the subset of the sample that was 'negative' by exome, they authors used RNA sequencing of the entire transcriptome from blood or fibroblasts, with some confirmations by RTPCR, of the disruption of mRNA due to rare DNA mutations. The work is somewhat confusing to read and in its presentation, but important points are contributed by this manuscript to the literature

*We regret not having made ourselves clear in the section surrounding WES-negative cases. Our goal here was to follow up on the “unbiased” breakdown of diseasecausing variants using the cohort of single locus-mapped families. If our prediction from that analysis were correct, we should see that most WES-negative cases were actually called “negative” at the interpretation rather than the capture stage because, at least in the case of recessive diseases, we had shown that the overwhelming majority are not caused by variants that are not typically captured by WES. Indeed, our reanalysis of these WES-negative cases did identify the causal variant in the majority, and the interpretation of the identified variants was greatly aided by the use of RNA analysis where applicable. In order to make this point clear, we have now added a new Figure 1 that shows the pipeline of the study.*

11- Overall the introduction should be shortened, and the authors should remove redundancies such as: "However, the anticipated attainment of the maximum diagnostic yield through whole genome sequencing (WGS) has not been realized. The minimal boost of diagnostic yield offered by WGS over WES suggests that the bottleneck is not in the capture/calling of the causal variants in the sequencing stage but rather in their interpretation [4, 5].

This notion is supported by studies showing the value of careful reinterpretation of "negative" WES and how misinterpreting the causal variants in WES is a major challenge that cannot be circumvented by WGS [5, 6]." These three sentences say the same thing and make the manuscript much more difficult to read, and examples can be found throughout the introduction, results and discussion.

*Thank you for another great suggestion! We have now shortened Introduction and removed redundancies. As for the above sentences, we hope the respected reviewer will appreciate the importance of gradually introducing the authors to the central issue of “Why are we not achieving a 100% diagnostic rate by WES? Is this because all negative cases are caused by variants in areas of the genome that are not captured by WES?”, which will be critical to the understanding of the logic of the study as we explained in our response to point #10. We hope that reading these sentences in light of the new edits will make them appear more coherent and less redundant.*

12- The references for the manuscript are not entirely appropriate. For instance, references 1-2 that are cited are not as relevant as the two below which make the same points but are published much earlier.

Clinical whole-exome sequencing for the diagnosis of mendelian disorders. Yang Y, Muzny DM, Reid JG, Bainbridge MN, Willis A, et al. N Engl J Med. 2013 Oct 17;369(16):1502-11. doi: 10.1056/NEJMoa1306555. Epub 2013 Oct 2. Clinical exome sequencing for genetic identification of rare Mendelian disorders. Lee H, Deignan JL, Dorrani N, Strom SP, Kantarci S, et al. JAMA. 2014 Nov 12;312(18):1880-7. doi: 10.1001/jama.2014.14604

*Thank you very much for the helpful suggestions. These two key papers are now appropriately cited.*

13- I would also add in the following references directly relevant and the authors should consider reasons for their high observation rate of disease causing DNA mutations that affect splicing relative to the prior work. Lee H, Huang AY, Wang LK, Yoon AJ, Renteria G, et al. Diagnostic utility of transcriptome sequencing for rare Mendelian diseases. Genet Med. 2019 Oct 14. doi: 10.1038/s41436-019-0672-1. [Epub ahead of print] PubMed PMID: 31607746.

*This and several other references that have just appeared very recently are now cited. It is important that we state the limited nature of our RNA-Seq analysis in this paper. After all, we have only conducted RNA-Seq analysis on the 6 cases whose phenotype could be mapped to single loci but had negative clinical WES. So in essence, this was a very small part of our paper compared to other papers that solely focused on the utility of RNA-Seq in the diagnosis of Mendelian conditions. Indeed, our study approaches the problem differently by trying to gain some fundamental knowledge about the patterns of contribution of transcript-deleterious variants to Mendelian diseases.*

14- Finally, the authors should remove genes and data from table S3 that include the proposed 'novel genes' that will be reported elsewhere, as it is not possible to evaluate in this manuscript in this manner.

*As the respected reviewer can appreciate, removing these cases will skew the results. Please allow us to list these novel findings below for your review (not sure if this will show using the text box in the website):*

*DG ID numberPhenotypeGene Mutation HGVS nomenclatureZygosityType of mutation 18DG0670Abnormal hair, nails, dyspigmentation and ichthyosisPERPNM_022121.4:c.466G>A:p.(Gly156Arg)HomozygousMissense 17DG0527Global developmental delay, hypotonia, epilepsy, postnatal microcephaly, strabismus and choreoathetosisYIF1BNM_001145463.1:c.177dupT; p.(Ala60Cysfs*13)HomozygousFrameshift indel 15DG1507Epilepsy and global developmental delayGLP1RNM_002062.3:c.402+3delG HomozygousTranscript-deleterious variantc 19DG0230Griscelli syndromeARID1BNM_020732.3:c.5683G>T:(p.Gly1895Trp)HomozygousMissense 16DG0559Joubert syndromeLRRC34NM_001172779:c.199A>T:p.(Lys67*)HomozygousNonsense 18DG0320Multiple congenital anomaliesWDR59NM_030581.3:c.2887G>A:p.(Gly963Arg)HomozygousMissense 17DG0872Hydrocephalus, agenesis of corpus callosum, macrocephalyDECR2NM_020664.4:c.149G>T:p.(Arg50Leu)HomozygousTranscriptdeleterious variantsb 13DG1688Short stature, microcephalySCUBE3 NM_001303136.2:c.2441T>C:p.(Ile814Thr)HomoygousMissense 14DG0835 Ehlers-Danlos syndrome (EDS)OLA1NM_013341.4:c.427C>T :p.(Arg143*)HomozygousNonsense 18DG0989Neonatal Adrenoleukodystrphy FAAH2NM_174912.3:c.1175G>A:p.(Trp392*)HomozygousMissense 19DG0509Undefined epileptic encephalopathyDALRD3NM_001276405.1:c.1251C>A:p.(Tyr417*)HomozygousNonse nse 18DG0669Microcephaly, Atrial septal defect, Ventricular septal defectC16orf96NM_001145011.1:c.2241+1G>THomozygousTranscript-deleterious variantsa 17DG0738Chronic interstitial kidney disease with small kidneys and borderline microcephaly (?Galloway-Mowat Syndrome)RBM47NM_019027:c.1416delC:p.(Tyr472*)HomozygousNonsense*

*Please note that we are now able to reveal PERP and LRRC34 because they were accepted for publication.*

*With many thanks!*

Reviewer #2: We are grateful to the respected reviewer for their positive remarks on the value of our study. We also sincerely thank the respected reviewer for their time and for very insightful comments. Below, please find our point-by-point response:

1)     The abstract is long and overly focuses on the large cohort. However, the cohort is not used for the majority of the novel features of the paper beyond describing the distribution of transcript-deleterious variants. The authors should focus on shortening the abstract and making clear the most important points.

*Excellent suggestion! The abstract has been edited to both shorten it and make it more focused as follows:*

*At least 50% of patients remain undiagnosed after whole-exome sequencing (WES), and the extent to which non-coding variants that are not captured by WES contribute to this fraction is unclear. Whole transcriptome sequencing (RNA-Seq) is a promising supplementary to WES, although empirical data on the contribution of RNA analysis to the diagnosis of Mendelian diseases on a large scale are scarce. Here, we describe our experience with “transcript-deleterious variants (TDVs)” based on a cohort of 5,647 families with suspected Mendelian diseases. We first interrogated all families for which the respective Mendelian phenotype could be mapped to a single locus to obtain an unbiased estimate of the contribution of TDVs at 18% (only 2.9% are not captured by WES). We then examined the entire cohort and found that TDVs account for 14% of all “solved” cases. RNA samples were available for 64%% of TDVs in our cohort so we can compare the results of RTPCR to in silico prediction. Definitive results from RTPCR could be obtained from blood-derived RNA for the overwhelming majority of variants (83.4%), and only a small minority (4.5%) failed analysis on both blood- and skin fibroblast-derived RNA samples (full concordance between the two sources of RNA was noted when the gene was expressed), which has important implications for the clinical application of RNA-Seq. We also show that RNA analysis could establish the diagnosis in ~13.54% of 155 patients who had received “negative” clinical WES reports. Finally, our data suggest a role for TDVs in modulating penetrance even in otherwise highly penetrant Mendelian disorders. Our results provide much needed empirical data for the impending implementation of diagnostic RNA-Seq in conjunction with genome sequencing.*

2)     Estimation of likely causal variants driven by structural variants is underestimated and not calculated. Many of these SVs may have transcript deleterious impacts and this may increase the percentages beyond 18%. Many of the 43% without a causal variant from the 154 select cases could be transcript-deleterious and driven by more complex variant types.

*The respected reviewer makes an excellent point here and we appreciate the opportunity to address it as follows. Based on our “unbiased” breakdown of disease causing variants in a cohort of 157 families whose diseases can be mapped to single loci (please note this is up from the previously submitted number of 156 because we were able to secure additional family members for one family, thus allowing us to map it a single locus), we know that SVs (defined here as deletions or insertions >50bp in size) only account for five of these 157. Of course, we are fully aware that our analysis is not generalizable since all families that mapped to a single locus had autosomal recessive phenotypes and we do not know what the equivalent percentage is for dominant disorders due to lack of a similarly unbiased cohort. Since the majority of the WES-negative families have phenotypes consistent with autosomal recessive inheritance, we suspect that SVs (including SVs with a transcript deleterious impact) will only account for a small fraction. Nonetheless, we have now honored the respected reviewer’s request and run chromosomal microarray analysis on these cases and have found none with structural variants. Please note that we are excited to report an update on the percentage of WES-negative cases that remained negative after reanalysis, down to 40% from our previously submitted 42.2% thanks to our continued reanalysis during the review process. All relevant percentages, tables and figures have been updated accordingly.*

3)     The alpha and beta scores seem largely uniformative for recovering candidate genes. The most impactful filter is the autozygome coordinate-filtering. The authors should comment more on this and how to successfully interpret these scores.

*We regret that the importance of the alpha and beta scores was not conveyed clearly in the original manuscript. These scores were used to measure the outlierness of genes in the cases and they played an important role in ranking and filtering candidate genes. For example, in undiagnosed case 16DG1048, the positional filtering based on a single locus reduced the candidates to 98 genes, and the alpha/beta score-based filtering was able to correctly pick PEX19 as the only candidate gene. So, although, as the respected reviewer correctly pointed it out, the coordinate-based filtering played a crucial role in narrowing down the causal gene search space, the score-based filtering also played an importance role in reducing the search space and correctly predicting the causal genes.*

*To better communicate the interpretation of the alpha and beta scores, we have added the following in the revised manuscript: “a high alpha score means that the abundance level of normal transcripts of a given gene is lower compared with a lower-end abundance of the same gene in the control set. Similarly, a high beta score means that the fraction of the normal transcripts of a given gene is lower compared with a lower end of the same gene in the control set.”*

4)     How do the alpha/beta scores compare to much simpler expression and splicing Z-scores?

*We appreciate this comment very much. As the respected reviewer may well be aware, commonly used approaches for the RNA-Seq-based causal gene prediction are to have separate pipelines to identify expression outliers and splicing outliers. In contrast, the approach we took with the alpha/beta scores uses a single pipeline with a single RNA-Seq quantification to identify outliers based on the abundance level of splicing-error-free transcripts. With this approach, for example, genes with high alpha and low beta scores can indicate expression outliers with low promoter activity or splicing outliers with aberrant transcripts degraded via NMD, while genes with high alpha and high beta scores can indicate splicing outliers with large fractions of expressed transcripts having splicing errors.*

*We would like to clarify that the main aim of the RNA-Seq-based causal gene prediction in the present study is to empirically test our hypothetical diagnostic yield of RNA-Seq. We believed that the positive prediction results from the six known cases served as evidence to use the causal gene prediction yield as a reasonable number for this purpose. And it is not our intention to claim that our RNA-Seq causal gene prediction pipeline outperforms existing approaches. Nevertheless, in this revision, we have included the results of causal gene prediction performance comparison against existing approaches (five splicing outlier-based and one expression outlier-based). A newly added supplementary table (Table S6) shows the comparison results. We found that our alpha/beta score-based approach outperformed the other approaches based on the prediction of the true causal genes and the filtering of candidate genes (i.e., the reduction of false positives).*

5)     A method called Outrider, AJHG, has been published for normalization of RNA-seq and detection of rare disease genes. Have the authors evaluated the use of this approach on their data?

*Thank you very much for introducing this paper to us. Our pipeline and OUTRIDER are very different approaches with different criteria for outlier calling. OUTRIDER, as with typical expression outlier detection methods, focuses on abundance levels of overall transcripts regardless of the nature of their function. On the other hand, our pipeline focuses on outlier calling based on the abundance level of splicing-error-free transcripts. For genes with noncoding regulatory variants which decrease the transcription rates or with splicing variants which activates NMD and quickly degrades a large fraction of aberrant transcripts, these two approaches may produce similar prediction results as the gene-level abundances of overall transcripts can call those genes as outliers. However, in general, we’d expect outlier calling results be different between these two different approaches. With that being said, we have used OUTRIDER on our data in a comparative study for this revision. Overall, we found that OUTRIDER produced a very small number of candidate genes and, of six cases, it was able to correctly predict the causal gene for just one case. The performance comparison results are summarized in a supplementary table S6. Our setup of the comparative analysis is described in a new supplementary material.*

6)     The abstract highlights a discussion of penetrance but this is not clearly provided in the results. Instead there are results about canonical splicing variants in disease genes with no phenotype and potential tissue-specificity of some of these effects. This should be removed from the abstract or discussed more clearly.

*In the previous submission, we did highlight several instances of non-penetrance as follows: “More concerning was the finding of canonical splicing variants in established disease genes with no resulting phenotype. For example, the variant NM_001172818.1:c.300+1G>A in PGM1, was identified in homozygosity in individuals with no phenotype, which explains its high population frequency. Similarly, we have identified an individual with ambiguous genitalia who is homozygous for LRP4:NM_002334:exon7:c.796+2T>C but lacks all features of established LRP4- related syndromes. On the other hand, the finding of ARHGAP31:NM_020754.2: c.539+1G>A in asymptomatic individuals despite its deleterious effect on splicing (confirmed by RTPCR) can be attributed to the fact that previously reported mutations in this gene were proposed to be gain-of-function. The SBDS founder variant is also worth highlighting since this is the most commonly reported variant in SchwachmanDiamond syndrome (SDS) and yet we identified it in homozygosity in at least three individuals who lack SDS features. Upon further investigation, we found that this is a leaky splicing variant and that all previously reported SDS patients were compound heterozygous for a more severe truncating variant (Table S5). Finally, we note the unusual result of normal RTPCR on a patient with Marfan syndrome and a de novo FBN1:NM_000138.4:c.6872-1G>A variant, which suggests that the effect of splicing may be tissue-specific.”*

*We now edit the leading sentence to read “More concerning was the finding of canonical splicing variants in established disease genes with no resulting phenotype i.e. non-penetrance.”*

*In addition, we now make the following edit in Discussion: Original “Fifth, although our study did not specifically aim to compare splicing to other classes of variants, we think that the examples we encountered with respect to the phenotypic expression of homozygous vs compound heterozygous regulatory variants is noteworthy.”*

*Revised “Fifth, we show several instances of abnormal splicing with no resulting phenotype as well as normal splicing with resulting phenotype for well-established disease genes. The apparent non-penetrance in the former scenario could be alternatively explained by a tissue-specific effect, which could also explain the latter scenario. Fortunately, these appear to be the exception; however, they are useful reminders of the expected limitation of RNA-Seq on clinically accessible samples. Sixth, although our study did not specifically aim to compare splicing to other classes of variants, we think that the examples we encountered with respect to the phenotypic expression of homozygous vs compound heterozygous regulatory variants are noteworthy.”*

7)     Many of the supplemental table legends were cut-off in the PDF. Please provide an index of tables and their legends as a separate page at the end of the manuscript.

*We sincerely apologize for the glitch. We have appealed to the editor to make all tables available to the respected reviewers as Excel files in addition to the rendered PDF format, which we did modify exactly as suggested by the respected reviewer.*

8)     The observations that the majority of transcript deleterious variants could be found between tissues is encouraging and an important observation. Is there more overlap of studied disease genes between tissues than expected by chance? Could this percentage be calculated directly from OMIM or other data?

Thank you very much for the encouraging comment. Indeed, we do believe that our ability to show *the deleterious effect of TDV in more than one tissue per variant is an important contribution. In fact, we are pleased to show that we have now increased the number of variants for which we were able to test more than one tissue to 15. However, we are aware that these are nonetheless small figures that do not allow us to make such bold statements as to calculate whether the overlap is more than expected by chance. As for the last question, we hope we had understood it correctly when we answer by saying that it is best addressed by future studies in which RNA-Seq is performed on multiple tissues per patient.*

9)     What allele-frequency filters were used, if any? Can the authors report the allele frequency distribution of their different classes of variants?

*We only considered variants with allele frequency of <0.001 in gnomAD and a local database of 2,379 ethnically matched exomes. We now provide the allele frequency for all the variants listed in Table S7.*

10)     Does existing phenotype information aid in recovering target genes? Approaches like AMELIE have been designed to help reduce the scope of target genes further. How would these data converge in their positional filtering? My concern is whether RNA-seq here gives anything more than this other type of approach.

*We would expect that disease phenotype information is very useful, especially for known disease genes, and we are developing a diagnostic tool which integrates heterogeneous data including disease phenotype data.*

*AMELIE uses a large corpus of literature to rank candidate genes. This type of knowledge-based approaches is complementary to RNA-Seq-based approaches. And, in particular for WES-negative cases, we believe that RNA-Seq-based approaches can play an important role to better interpret the functional consequences of variants of unknown significance and search for novel disease-causing variants. To address this comment with empirical evidence, we have used AMELIE to rank candidate genes selected using our positional filtering. The results are summarized in the table below (not sure if this will show using the text box in the website).*

*Case IDCausal gene score rankNo. of genes with scores within ROHHPO terms 10DG0840134HP:0000750; HP:0001260; HP:0001508; HP:0002066; HP:0000824; HP:0001250; HP:0001257 11DG01657616HP:0001999; HP:0001317; HP:0000528; HP:0011483; HP:0002804; HP:0001321; HP:0003236; HP:0001290; HP:0001339; HP:0002162; HP:0000568; HP:0000278; HP:0010537 15DG215411HP:0001167; HP:0001760; HP:0003982; HP:0004977; HP:0001776; HP:0000476; HP:0100257; HP:0003038; HP:0000774; HP:0002089; HP:0005736; HP:0000474; HP:0005257; HP:0002119 16DG1048448HP:0000971; HP:0000750; HP:0002572; HP:0001371; HP:0001263; HP:0001347; HP:0000252; HP:0001250; HP:0002218; HP:0002510; HP:0001257 16DG16201178HP:0001999; HP:0430026; HP:0000618; HP:0000276; HP:0008551; HP:0011002; HP:0002757; HP:0005590 15DG2234N/A14HP:0002500; HP:0001249; HP:0000252*

*Our results show evidence that, although knowledge-based approaches can filter out false positives further, their usefulness may be limited for novel cases.*

11)     The paper focuses mostly on homozygous recessive alleles and would benefit from making this clearer. There are approaches using RNA-seq such as ASE-based analyses that are not possible to leverage here but that may yield more traction in other areas. Please see the recent paper by Mohammadi in Science, 2019 for ANEVA.

*Thank you very much for introducing this paper to us. Yes, as the respected reviewer indicated, we have used positional filtering based on ROH, and we do focus on homozygous recessive alleles in the RNA-Seq-based analysis. In the revised manuscript, we have clarified this point by changing a sentence in the Results section as follows: “To test this empirically, we set out to investigate six cases whose autosomal recessive Mendelian phenotypes map to single loci.”*

12)   The authors need a more specific title to clarify their works impact. The title is too general particularly with existing studies in this area.

*We appreciate the comment. To honor the respected reviewer’s suggestion, we now changed our title from “RNA-based diagnosis of Mendelian disorders” to “Analysis of Transcript-Deleterious Variants in Mendelian Disorders: Implications for RNA-based Diagnostics”.*

*With many thanks!*

Reviewer #3: *We thank the respected reviewer for the thorough review and would like to respond to the points they raised as follows:*

1- Overall, the manuscript is hard to follow, lacks a clear message, is confusing and misleading.

*We regret that the reviewer found our manuscript hard to follow, lack a clear message, confusing and misleading. Part of the challenge in this paper is our attempt to present a large amount of data in a single paper. In essence, our paper has several parts that should have been presented in the following logical order:*

*a) Exploiting the special structure of our study population to address a critical question in Mendelian genetics. There is a growing concern that WES (whole-exome sequencing) may have reached its diagnostic limit and that WGS (whole-genome sequencing) should be implemented instead to detect non-coding variants that are presumed to underlie Mendelian cases that remain negative after WES. However, the question of whether most WES-negative cases are indeed caused by non-coding variants that are not captured by WES must be addressed empirically by answering another question: what is the proportion of Mendelian diseases-causing variants that reside within the uncaptured part of the genome during WES? This is a very difficult question to address because the denominator and enumerator are under a very strong negative bias. Indeed, investigators are much more likely to identify variants in the coding and flanking non-coding part of the genome than in deeper parts of the genome. In order to circumvent this bias, we propose to resort to the “unbiased” nature of positional mapping where a phenotype is mapped to a single locus regardless of the nature of the causal variant. In other words, easily identifiable mutations and challenging deep intronic mutations are just as amenable to positional mapping. So if we focus on families in which Mendelian phenotypes can be mapped to single loci (this is typically possible when we have sufficient numbers of affected and unaffected members in a given family), we can have an “unbiased” breakdown of the classes of disease-causing variants in humans. This is indeed what we achieved in this study by analyzing 157 families in which we had been able to map various Mendelian phenotypes to single loci, the largest collection of single locus mapped phenotypes in any study to date to the best of our knowledge.*

*b) Equipped with potential answer to the question in (a), we then turned to our large cohort of patients with Mendelian disorders in whom we did identify a likely causal variant over the lifespan of our lab. We selectively extracted transcript-deleterious variants (TDVs) from that large cohort to estimate their overall contribution. The answer we obtained from (a) is very helpful to gauge the answer obtained in (b). Without (a) we could be criticized that the percentage obtained in (b) is a gross underestimate of TDVs since many are difficult to identify. However, we have repeatedly shown that homozygosity-guided analysis of cases greatly aids in the identification of even the most challenging of variants. Indeed, the estimate we had from (b) is only slightly lower than (a).*

*c) We exploited the availability of RNA material for the majority of TDVs in our cohort, we empirically tested the effect of these variants on RNA. This exercise served two mains purposes: i) it allowed us to document the actual effect of these variants on RNA instead of relying on in silico prediction such that we were able to comment on the limitations of the commonly used in silico tools in interpreting novel variants, ii) test whether clinically accessible tissues (primarily blood followed by skin fibroblasts) can recapitulate the deleterious effect of TDVs on RNA even though the overwhelming majority of these phenotypes are neurological, which has important implications for the application of RNA-Seq in diagnostics.*

*d) We reanalyzed a large cohort of cases that were called “negative” by clinical WES and affirmed the practical implications of points a, b and c in that we found that most of these cases were not negative because of technical limitation of WES to capture deep intronic mutations, but rather due to interpretation challenges. By quantifying how many of these cases are caused by TDVs, we are able to inform the development of protocols on combining the use of WES with RNA-Seq. In view of the above, we respectfully disagree with the reviewer that the paper lacks a clear message. We also hope that their comment about it being “misleading” and “confusing” are merely reflective of our failure to communicate the data more effectively in the previous submission. We have taken their feedback to heart and implemented many changes that will be outlined as we respond to the specific points below.*

2- It generally lacks systematic assessments, justifications of the claims, and comparisons to the state-of-the-art.

*We respectfully disagree with the reviewer that our paper lacks systematic assessments. We have indeed systematically surveyed every single causal variant our lab has ever identified in its lifespan of more than 12yrs and covering more than 5,500 families with a very wide range of Mendelian disorders. We are not aware of any similarly systematic survey of TDVs in a single paper. Similarly, we are not aware of any similarly systematic analysis of TDVs using RNA from patients, cataloging the results and comparing them to the state-of-the-art in silico tools. The unbiased and systematic assessment of every single family in our cohort that maps to a single critical locus regardless of the ascertainment phenotype is the largest to date. Finally, our systematic analysis of all WES-negative cases referred to our lab is one of the largest to date, second only to the study PMID: 31231135 that analyzed 313 families. For each part of our paper, we were careful to only make claims that are backed by empirical data.*

3- The two main figures are descriptive and lack a conclusive message

*Thank you very much for the comment. In compliance with the suggestion by the respected reviewer, we have made the following changes to the figures:*

*Figure 1: A flow chart of the entire study with its different parts.*

*Figure 2: The new Figure 2 lists the “unbiased” breakdown of all causal variants in a large cohort of families that map to a single locus. The mains message of this figure shows how the overwhelming majority of these variants should be picked up by exome sequencing.*

*Figure 3: This is the old Figure 1. We hope the respected reviewer will reconsider the importance of this figure in which we show a complete list of TDVs and their breakdown by category. We now added the results of RTPCR to comply with another suggestion by the same reviewer (see below). Figure 4: This figure summarizes the results of our reanalysis of “WES-negative” cases.*

*Figure S1: The new Figure S1 summarizes the results of RNA-Seq both on positive controls as well as those that were unknown.*

4) It is hard to understand what the key point of the paper is. The abstract is extremely long without a clear structure delineating the addressed question, main claims, and implication.

*We regret that the respected reviewer found it hard to understand the key point of the paper. Perhaps our attempt to include a large amount of data in a single paper is to blame, but we take this criticism positively and make a new attempt to communicate our results as clearly as possible. To this end, we should have arranged the flow the paper to highlight the following important messages:*

*a) Exploiting the special structure of our study population to address a critical question in Mendelian genetics. There is a growing concern that WES (whole-exome sequencing) may have reached its diagnostic limit and that WGS (whole-genome sequencing) should be implemented instead to detect non-coding variants that are presumed to underlie Mendelian cases that remain negative after WES. However, the question of whether most WES-negative cases are indeed caused by non-coding variants that are not captured by WES must be addressed empirically by answering another question: what is the proportion of Mendelian diseases-causing variants that reside within the uncaptured part of the genome during WES? This is a very difficult question to address because the denominator and enumerator are under a very strong negative bias. Indeed, investigators are much more likely to identify variants in the coding and flanking non-coding part of the genome than in deeper parts of the genome. In order to circumvent this bias, we propose to resort to the “unbiased” nature of positional mapping where a phenotype is mapped to a single locus regardless of the nature of the causal variant. In other words, easily identifiable mutations and challenging deep intronic mutations are just as amenable to positional mapping. So if we focus on families in which Mendelian phenotypes can be mapped to single loci (this is typically possible when we have sufficient numbers of affected and unaffected members in a given family), we can have an “unbiased” breakdown of the classes of disease-causing variants in humans. This is indeed what we achieved in this study by analyzing 157 families in which we had been able to map various Mendelian phenotypes to single loci, the largest collection of single locus mapped phenotypes in any study to date to the best of our knowledge.*

*b) Equipped with potential answer to the question in (a), we then turned to our large cohort of patients with Mendelian disorders in whom we did identify a likely causal variant over the lifespan of our lab. We selectively extracted transcript-deleterious variants (TDVs) from that large cohort to estimate their overall contribution. The answer we obtained from (a) is very helpful to gauge the answer obtained in (b). Without (a) we could be criticized that the percentage obtained in (b) is a gross underestimate of TDVs since many are difficult to identify. However, we have repeatedly shown that homozygosity-guided analysis of cases greatly aids in the identification of even the most challenging of variants. Indeed, the estimate we had from (b) is only slightly lower than (a).*

*c) We exploited the availability of RNA material for the majority of TDVs in our cohort, we empirically tested the effect of these variants on RNA. This exercise served two mains purposes: i) it allowed us to document the actual effect of these variants on RNA instead of relying on in silico prediction such that we were able to comment on the limitations of the commonly used in silico tools in interpreting novel variants, ii) test whether clinically accessible tissues (primarily blood followed by skin fibroblasts) can recapitulate the deleterious effect of TDVs on RNA even though the overwhelming majority of these phenotypes are neurological, which has important implications for the application of RNA-Seq in diagnostics.*

*d) We reanalyzed a large cohort of cases that were called “negative” by clinical WES and affirmed the practical implications of points a, b and c in that we found that most of these cases were not negative because of technical limitation of WES to capture deep intronic mutations, but rather due to interpretation challenges. By quantifying how many of these cases are caused by TDVs, we are able to inform the development of protocols on combining the use of WES with RNA-Seq.*

*The title, abstract, main text and figures have all been restructured to reflect this order, which we hope the respected reviewer more effectively communicates an important set of results to the community.*

5) The title "RNA-based diagnosis of Mendelian disorders" suggests that the paper will discuss cases that were solved with the aid of RNA-Seq. However, RNA-Seq was performed on 6 exome negative cases, out of which they were able to solve 1. The manuscript is instead articulated around assessing the association of their proposed "transcript-deleterious variants" with disease, and to some extent, with aberrant expression and aberrant splice isoform usage of the respective genes.

*We never intended for our title to mislead the readers into thinking that our paper is about cases solved by RNA-Seq. Instead, we aimed at highlighting the central message of the paper, which is to expand the current knowledge concerning what to expect when one uses RNA to diagnose a Mendelian condition. To honor the respected reviewer’s suggestion, we have now changed the title from “RNA-based diagnosis of Mendelian disorders” to “Analysis of Transcript-Deleterious Variants in Mendelian Disorders: Implications for RNA-based Diagnostics”.*

6) The concept or transcript-deleterious variant, which is central to the paper, is unclear. The abstract defines those variants as "variants likely to cause their phenotypes by interfering with the normal transcription of the respective gene". This is wrong. Transcription refers to RNA synthesis. In contrast, the variants considered by the authors include variants affecting RNA processing (in particular splicing). Literally, a "transcript-deleterious variant" should refer to any variant deleterious for the transcript. However, they excluded protein-truncating coding variants (according to the abstract) although these variants potentially affect RNA stability by triggering nonsense mediated decay. Moreover, the variants are defined based on in silico criteria. Should not they be therefore designated as "potentially deleterious"? An alternative definition could be "transcript-encoded variants" but would then variants located outside the processed RNA such as intronic variants be considered? Without a clear conceptual definition of this central notion, the study falls apart.

*We respectfully disagree with the reviewer’s assertion that we did not provide a clear conceptual definition of what transcript-deleterious variants (TDVs) are. Each class was meticulously defined in the Materials and Methods and the numerous examples of each category as well as their empirically tested RNA outcome are listed in extensive tables. As for the semantics of “transcription” vs “splicing”, we now change the overall definition from “variants likely to cause their phenotypes by interfering with the normal transcription of the respective gene” to “variants likely to cause their phenotypes by interfering with the final transcript of the respective gene” to satisfy all categories. As for logic behind excluding nonsense variants even though they may exert their effect at the level of the final transcript via NMD, we now make this clear in Materials and Methods as follows: “Although nonsense variants may exert their pathogenic effect at the level of the final transcript via NMD, we have chosen to exclude them because it is very difficult to disentangle their effect on protein from that on RNA”.*

7) The executive definition of the transcript-deleterious variants is also unclear. In particular, what are "noncanonical splice site intronic variants" (Class c)? Also, which algorithm has been used to identify "promoter/enhancer elements" (Class f)?

*We had hoped that by defining “canonical” for class a (the first and last 2bp of each intron), we do not have to define “non-canonical” for class c and avoid redundancy. To honor the respected reviewer’s suggestion, we now define class c as “non-canonical splice site intronic variants i.e. other than the first and last 2bp of an intron”. We did not use any algorithm in defining promoters and enhancers because all the identified variants therein were called based on previously established and published promoters and enhancers in the literature.*

8) Variants were classified as candidate transcript-deleterious if they were compatible with pathogenicity potential in terms of frequency and segregation. However, there are no details of the cutoffs used.

*Excellent point! We now update the tables to include allele frequency data based on gnomAD as well as ethnically matched exomes as we now explain in the revised Materials and Methods. There is no cutoff for segregation because we simply did not report any variant that did not fully segregate with the disease within the respective family.*

9) The authors apply 4 different prediction tools to the 6 classes of variants that they define. They find that 7% of "class a" variants have inconsistent predictions among the 4 methods, but from the other 5 classes combined, as much as 44.8% of the variants have inconsistent predictions. Aggregating the results of the 5 groups is misleading. Moreover, it is unclear what the goal was when applying the methods. Splice-AI, TRAP, S-CAP are splicing predictors whereas CADD is a general-purpose score. This should be explained and taken into account in the interpretation of the results.

*We regret not having this point more clear. Of the six different categories, category a is by far the best understood, and this is why it is the only listed in the ACMG guidelines as PVS1. Our point in comparing category a vs all others combined was not meant to mislead, but simply to show the irony that our current tools perform indeed very well for this obvious category but not so well for the ones where we need these tools most. To honor the respected reviewer’s suggestion, we now do list the degree of agreement for each of the categories before we make that comparison.*

10) It is very difficult to follow the sample number breakdown (the main text is as confusing as the abstract). An overview diagram of the workflow showing the number of samples entering each stage (WES, RNA-seq, RT-PCR), the numbers of solved and unsolved, the numbers of affected and unaffected, would be helpful. Also, the authors should make a clear distinction between the number of families, the number of individuals, and the number of affected individuals.

*Excellent suggestion! We now honor the respected reviewer’s suggestion and make a new Figure 1 that provides an overview of the workflow. We believe it will be extremely confusing to go back and forth between families and number of affected members and this is why our reference has always been to families unless otherwise specified. Nonetheless, we now update the tables to indicate the total number of affected individuals.*

11) For a better understanding of the data, more details should be provided about the 5,647 families with suspected Mendelian phenotypes. Are these trios? Are these consanguineous families? Is there WES for all? How many individuals? What fraction of these families show autosomal recessive mode of inheritance?

*We do believe that what really matters to the readers from this cohort is the percentage of TDVs from among the solved families. Nonetheless, we now provide a brief summary that describes these families in terms of the percentage of consanguinity, simplex vs multiplex and recessive vs. dominant vs X-linked.*

12) Based on what criteria "a likely causal variant was identified in 2,429 of these families"? How is the functional relationship between genotype and phenotype assessed? In fact, there are only 156 families where Homozygosity track leads to a single locus, it is thus unclear how "likely causal" the variants of these 2,429 families are.

*We are a bit perplexed by both comments. Calling a likely causal variant has been fully standardized in recent years thanks to the ACMG guidelines which we do cite in our Materials and Methods. Similarly, one need not perform any mapping at all, let alone mapping to a single critical locus, to call a likely causal variant as such.*

13) Analysis of RNA-seq data is based on ref. 26, which is a non-peer-reviewed preprint of some of the authors. This preprint is poorly written. It is supposed to increase the sensitivity to detect genes with an aberrant splicing pattern but no assessment for that claim is provided. The only benchmark is a clustering of GTEx samples which is claimed to be more accurate than based on TPM. Clustering is not a piece of evidence for improved detection of aberrant splicing events. No ROC-curve nor precision-recall against splicing outlier detection methods (pipelines described by Cummings et al. /Gonorazky et al., Kremer et al, and Fresard et al.) is given. How is the overlap? Is there evidence for higher enrichment of rare variants or diseasecausing variants. It would be also interesting to compare it to SPOT (Ferraro et al, bioRxiv, https://www.biorxiv.org/content/10.1101/786053v1, online prior to the submission of this paper). Moreover, RNA-seq can lead to aberrant levels, aberrant splicing and mono-allelic expression (Kremer et al. Nat. communs, 2017). Only aberrant splicing is assessed with Omega as far as I understand. This should be clarified.

*We would like to thank the respected reviewer for taking the time to read our bioRxiv preprint for the omega quantification and giving us useful feedback. To address these multifaceted comments, we (i) first clarify what omega is, what it is not, and how it can be used to detect outliers based on our criteria, (ii) clarify the performance assessment we used in the bioRxiv preprint, (iii) clarify our aim of the RNA-Seq-based analysis in the present study, and (iv) finally describe our causal gene prediction performance comparison analysis which was conducted in response to the feedback.*

*(i) Omega is an RNA-Seq quantification method with a between-sample normalization which estimates the gene-level abundance of splicing-error-free transcripts. That is, unlike cluster-level PSI or other normalized splicing frequency quantification, omega does not quantify RNA-Seq data at the level of splicing events, but instead, omega represents genes by scaler values like gene-level TPM and RPKM. Thus, on the contrary to the respected reviewer’s notion, omega does not seek to detect aberrant splicing events. Because the omega quantification, as is, cannot be used for analysis of outlier genes, it requires downstream analysis to standardize the gene-level values and score them using the control set for outlier calling in the case set --- just like what we did in the present study. In our alpha/beta score-based outlier detection approach, a high alpha score means that the abundance level of normal transcripts of a given gene is lower compared with a lower-end abundance of the same gene in the control set. Similarly, a high beta score means that the fraction of the normal transcripts of a given gene is lower compared with a lower end of the same gene in the control set. Thus, for example, genes with high alpha and low beta scores can indicate expression outliers with low promoter activity or splicing outliers with aberrant transcripts degraded via NMD, while genes with high alpha and high beta scores can indicate splicing outliers with large fractions of transcripts escaping NMD. So, unlike commonly used approaches to have separate pipelines to detect expression outliers and splicing outliers, we can use a single pipeline with omega-based scoring to detect these two types of outliers. We are developing a diagnostic tool which integrates heterogeneous data sources, and we hope to show more comprehensive results of omega-based approach in the near future.*

*(ii) The clustering of GTEx samples that the respected reviewer referred to is a supplementary figure (Fig S1), and it was not used to show evidence for improved detection of aberrant splicing events as the respected reviewer indicated. As described in the Results section, this analysis was used solely to show evidence that the abundance of splicing-error-free transcripts, rather than the overall transcripts, can capture the phenotypic state of the cell. The performance evaluation results were shown in Figure 2 and Table 1 in the preprint. The results in Figure 2a show the number of candidate genes with the indication of the inclusion of the true causal genes over various threshold values and these can be interpreted as, for example, precision or false discovery rate over different threshold values. Table 1 shows the ranking of each causal gene based on the same scoring of the two quantification methods (TPM and omega).*

*Although the respected reviewer suggested the use of ROC curve or precision-recall against splicing outlier detection methods, objective comparison and performance evaluation of such heterogeneous outlier detection methods would be very difficult in our setting for several reasons. Firstly, each method has different criteria for the definition of outliers. Secondly, since we used real RNA-Seq datasets, they do not have the ground truth to classify outlier genes and non-outlier genes. Only information we had was the true causal gene for each case, and these causal genes may not be considered as outliers based on some criteria. The only way to have the true outlier and non-outlier gene sets is to resort to the use of simulation. However, this simulation would still require a specific criterion to define outliers. And whatever methods that use closest criteria for this outlier definition would be expected to perform well. Nevertheless, we have performed comparative causal gene prediction analysis using various methods that the respected reviewer suggested. We will describe more about this in (iv).*

*(iii) We would like to clarify that the main aim of the RNA-Seq-based causal gene prediction in the present study is to empirically test our hypothetical diagnostic yield of RNA-Seq. And it is not our intention to claim that our RNA-Seq causal gene prediction pipeline outperforms existing approaches. We believed that the positive prediction results from the six known cases served as evidence to use the causal gene prediction yield as a reasonable number for this purpose.*

*(iv) To honor the respected reviewer’s suggestion to compare our approach against existing approaches, we performed comparative causal gene prediction analysis using six existing methods (the splicing outlier detection approach used by Kremer et al., a leafcutter-based splicing outlier approach, SPOT which was used by Ferraro et al., the splicing outlier detection approach developed by Cummings et al., the splicing outlier approach used by Gonorazky et al., and OUTRIDER which was developed by Brechtmann et al.). Because the purpose of this comparison was to evaluate the genelevel prediction accuracy, we called the prediction of a splicing outlier method correct if its prediction included any splicing outlier within the true causal gene. The datasets we used for this comparison were the five known cases and the case in which KCTD3 was identified as the causal gene. For each case, we used a set of coding genes selected with our positional filtering as the initial potential candidates and evaluated the prediction performance by analyzing the correct prediction of the causal gene as well as the number of candidate genes. Our setup of the comparative analysis is described in a new supplementary material. A newly added supplementary table (Table S6) shows the comparison results. We found that our alpha/beta score-based approach outperformed the other approaches based both on the prediction of the true causal genes and the filtering of candidate genes (i.e., precision).*

14) The second criterion for a gene to be considered as an RNA candidate is that it must have the lowest alpha_g value out of all the other patients. Therefore, each gene can be an outlier of at most one patient, which even in rare diseases is not true. It would be good to know how many candidates were found in total.

*The respected reviewer is absolutely correct in that this filtering cannot be applied in a general setting. However, the reason why we applied this filtering criterion in our specific case is because we had in total 11 RNA-Seq datasets (4 in fibroblasts and 7 in LCL) from RNA samples with RIN > 8.5. These 11 cases exhibited different disease phenotypes, which allowed us to assume that the causal genes are different among them. And it is because of this small number of RNA-Seq datasets that we were able to use this very strict filtering criterion. The number of candidate genes selected prior to applying the positional-based filtering is shown in the Results section. Our alpha/beta-based filtering was able to reduce the search space from ~19,000 genes to ~100 genes in most cases.*

*To alleviate potential confusions, we have added the following sentence right after the statement for criterion 2: “Note that criterion 2 is based on 11 RNA-Seq datasets from RNA samples with RIN > 8.5 (4 from fibroblasts and 7 from LCL) and that this criterion was set specifically for comparison based on a small number of patients.” We also corrected a typographical error in criterion 2 and changed alpha_g to omega_g.*

15) The abstract states that the RNA analysis could establish a diagnosis in ~13% of 155 patients with exome negative results. However, the main text refers to 154 patients out of which 57% were solved after re-analysis of WES and 18% of which harboured a transcript-deleterious variant. The ~13% of the abstract is never mentioned in the text. On the contrary, from the text (and Fig 2B which is a pie chart with ⅙ colored), it seems that RNA-Seq helped solving 1 sample (Fig 2C) out of 6 tested.

*We appreciate the opportunity to clarify this apparent contradiction. Reanalysis of the 155WES-negative cases did indeed reveal a contribution of 22.7% of TDVs, but RNA analysis was required to establish the cause in only 13.54%. We agree that this is confusing so the relevant sections were edited accordingly. Please note that this has nothing to do with the RNA-Seq results.*

16) There is no data or quantification provided for the RT-PCR measurements. A figure providing the number of samples and results would be helpful. The abstract states that definitive results from RT-PCR could be obtained for 83.4% variants, but this is not shown in Results.

*Excellent suggestion! Figure 3 has been prepared accordingly. We also update Results to indicate the percentage of samples for which RTPCR provided definitive results.*

17) The discussion states that the autozygome coordinates filter reduces the search space by a factor of 300. However, this number is never mentioned throughout the manuscript. Moreover, expression or splicing outliers detection followed by false discovery rate cutoffs give a handful of candidates genes on RNA-seq and thus this filter might not be needed with state-of-the-art methods (see Kremer et al, Nat communs. 2017; Brechtmann et al. AJHG 2018 -- there is also a recent preprint Mertes et al. biorxiv 2019).

*Regarding the question about the reduction of search space by a factor of 300, we believe the respected reviewer is referring to the following sentence in the Discussion section: “For example, we show in this study that the use of autozygome coordinates drastically reduces the search space in RNA-Seq by up to a factor of 300.” This number was based on the results of 15DG2154, and it was mentioned in the subsection called RNA as a Tool to Solve “Negative” WES Cases as follows: “The RNA-Seq-based prediction generated 324 candidates. Among them, DONSON was ranked 200th on the alpha score and 281st on the beta score. With the autozygome coordinate-based filtering, DONSON was found to be the only candidate.” That is, with the alpha/beta-based filtering, we had a list of 324 candidate genes. And with the positional filtering, we were able to correctly pick DONSON as the only candidate. Thus, we were able to reduce the search space by up to a factor of 300. To address the other point that the respected reviewer mentioned in this comment, we would like to first reemphasize that the aim of the RNA-Seq-based analysis in the present study is to empirically test our hypothetical diagnostic yield of RNA-Seq. And this requires the use of positional filtering based on autozygome coordinates as we discussed in the manuscript. Although we are developing a diagnostic tool which integrates heterogenous data, it is out of scope to comprehensively evaluate the accuracy performance of various computational diagnostics tools in this study. Nevertheless, we have compared our alpha/beta-based approach with existing RNASeq-based approaches including the splicing outlier detection approach by Cumming et al. and OUTRIDER which was developed by Brechtmann et al. We found that, although OUTRIDER generated very small lists of candidate genes, as the respected reviewers predicted, it was only able to correctly predict one case out of six cases. As for performance results of the splicing outlier detection method by Kremer et al., on the contrary to the respected reviewer’s prediction, we found that this approach generated a very large number of candidate genes, suggesting that it would benefit from the use of additional sets of filtering.*

18) For reproducibility, the source code should be shared. Most crucial is the source code to classify variants in either of the categories a-f.

*We have uploaded the source code as well as necessary datasets to reproduce our RNA-Seq-based analysis results to GitHub (https://github.com/hkuwahara/RNASeqCausalGenePrediction). The classification of variants was manually done using our in-house database.*

19) Bottom of page 13: "A likely causal variant was subsequently identified in 57%." What does this mean? What are the criteria applied? Howe many variants these criteria yield per sample?

*The respected reviewer is kindly referred to our response to point #12. 20) It is unclear how Table 1 is established and why these represent solved cases. I picked up one example. DCDC2 is listed for patients with bile diseases (cholastic) but DCDC2 is not expressed in liver and has been so far associated with developmental dyslexia, which does not appear to be related to bile. This is just one example. For every gene, the relationship between presented phenotypes and gene function should be explained otherwise the causality seems dubious.*

*We must state that we are perplexed by this comment. DCDCD2 is a very well established disease gene for cholestasis and this link is both well published and listed in OMIM. We do not understand why the respected reviewer is questioning our finding of a clearly pathogenic variant in this gene. Similarly, all genes listed are either well established disease genes in OMIM or have at least the link with the disease published. The only exception is the handful of novel genes that we do not think is appropriate to reveal in this study and these are clearly labeled as “novel”. 21) P. 14 "These include a very deep (+335) variant in ABCB4 causing cholestatic disease in several affected members of an extended family" Why not with all affected members? Pedigrees must be provided to assess the reliability of the disease association claims.*

*The sentence has been The sentence has been revised to read “These include a very deep (+335) variant in ABCB4 causing cholestatic disease in all available affected members of an extended family (see Figure S2)”. A new Figure S2 has been added to satisfy the respected reviewer’s request.*

22) P.19 "Our results show that only 9.5% of these genes were not expressed in either RNA source." This claim should be substantiated with the number of genes reported per tissue, Venn diagram, and Method details provided. It feels rather low but it is all about a threshold issue. This statement is representative of the entire manuscript where figures are provided without point of reference, ways to assess the reliability of the number, details in the procedure, and comparison with the literature. Also, this falls short. Investigation of GTEx tissues could corroborate further this hypothesis. Have these genes or exons indeed some tissue-specificity? If so, is the tissue / the affected tissue or related to the phenotype?

*While our paper was being reviewed, a new paper just came out that is in full agreement with our conclusion that the overwhelming majority of neurodevelopmental genes are indeed expressed in blood derived cell lines (see PMID: 31911672). Thus, we do not believe that our result is an outlier. It is not clear to us what the respected reviewer meant by “this claim should be substantiated with the number of genes reported per tissue” because every single variant that we tested is listed in table S3 along with all the details concerning the source of RNA and the result of RTPCR. Furthermore, we now show that GTEx is an agreement with the 9.5% of genes not being expressed in blood or skin. Mapping individual exons for tissue-specific expression is beyond the scope of this work.*

23) Abstract and p. 7: the term "premature truncating coding variants" does not exist. It is either "protein-truncating variant" or "premature termination codon".

*Nonsense variants are indeed prematurely truncating coding variants. Nonetheless, we honor the respected reviewer’s request and change to “protein-truncating variants”.*

24) p. 8 "invariant splicing codes": this term is unclear and does not exist in the literature.

*Changed to “consensus splicing codes”.*

25) The 4 tested prediction tools are not cited.

*Thank you for alerting us to this inadvertent oversight, which is now addressed.*

26) Gonorazky et al. AJHG 2019 should be referred to and compared to.

*Cited and compared to as suggested.*

27) Aicher et al biorxiv https://www.biorxiv.org/content/10.1101/727586v1 should be discussed.

*We appreciate this suggestion. We have cited this paper for the following sentence in the Discussion section: “These factors make the use of RNA-Seq in clinical diagnostics challenging and highlight the need for empirical data e.g. mapping splicing variations in clinically-accessible tissue, that inform the development of computational tools that unlock the full potential of this technology.”*

28) P. 13 "Table S3 shows that many of these cases harbored a likely deleterious variant in a gene that was novel at the time". What does "the gene was novel" mean? Not known? Not annotated? Not associated with any disease? Not associated with this disease?

*It is a standard nomenclature in human genetics to refer to a gene with no established disease phenotype as novel. The message here is that the causal variant was missed because the gene was still novel i.e. not known to be associated with a Mendelian disease (GWAS associations don’t count in this context) at the time of clinical WES. This is now clarified in the table legend to avoid confusion.*

29) P. 18 "Class b should be lumped with class a": there is probably a selection bias if the variants were selected to associate with the disease. Actually, effects of splice dinucleotides variants (which define splice sites) on splicing are stronger than effects of variants on the two flanking nucleotides (which have a strong quantitative effect but are not splice site defining).

*We are perplexed by the suggestion to lump classes a and b together. These are very different classes from a clinical molecular laboratory perspective and combining them is not any more logical than combining other two categories of TDVs*.

30) In general, the Materials and Methods section lack content.

*This is a very general statement that is hard to respond to. However, we hope the respected reviewer will appreciate the updated Materials and Methods section in response to the various helpful suggestions made by this and other respected reviewers.*

*With many thanks!*

**Second round of review**

**Reviewer 2**

The authors have done a great job on improving their manuscript. I have gone through both mine and Reviewer 1's comments and feel that they were appropriately addressed as well as the changes to the manuscript. I will note that some changes in the manuscript looked like there might be repetitive text but this might be an issue with track changes. For example "To visualize splicing events, BAM files were first converted into the hg19 coordinate using CrossMap [30] and Integrative Genomic Viewer [31] was used. We have also attempted to compare our method to previously published methods as explained in Supplemental file 1.To visualize splicing events, BAM files were first converted into the hg19 coordinate using CrossMap [27] and Integrative Genomic Viewer [28] was used."
Furthermore, some of the reference changes Reviewer 1 requested I did not see changed in the new reference list. I am ok though with the authors not adding these intro papers or feeling the need to cite other RNA-seq diagnostic papers that have come out more recently. There are indeed several more beyond Reviewer 1's set where gene expression has aided a Mendelian diagnosis and a comprehensive review doesn't seem necessary here (or indeed the authors could just cite a review of this area along with their current citations). Overall, I think this paper makes a good contribution to the field providing a potential upper bound for mendelian diagnoses of TDVs with RNA-seq in autosomal recessive diseases.

Minor comment: Please add a breakdown of disease categories (neuro, immune, cancer, etc) to a main figure.

**Reviewer 3**

Overall, the authors have addressed the core issue. The manuscript is indeed much better organized now.  The new title reflects the aim of the study in a clearer way. The abstract and the new figures are very helpful. Because the focus is not on RNA-seq analysis we strongly suggest the authors to uncouple this paper from their method paper on biorxiv, by ensuring that the main message holds independently of the RNA-seq analysis method.
I acknowledge that the core idea of the manuscript is interesting, useful, and that the size of the dataset is impressive. However, I remain with the uncomfortable impression of a lack of rigour as reflected by the lack of coherence between the response and the manuscript and the limited possibilities for readers to reproduce the results and methods.

As new minor points:
- Abstract  “At least 50% of patients remain undiagnosed”. Which patients should be specified, probably  “At least 50% of patients with a suspected Mendelian disorder”.
- Figure 4 is referred to before Figure 2. Also, Figure S2 is mentioned before Figure S1.

I am giving a point-by-point response to the remaining open points below.

3- The two main figures are descriptive and lack a conclusive message.
Thank you very much for the comment. In compliance with the suggestion by the respected reviewer, we have made the following changes to the figures:
Figure 1: A flow chart of the entire study with its different parts.
Figure 2: The new Figure 2 lists the “unbiased” breakdown of all causal variants in a large cohort of families that map to a single locus. The mains message of this figure shows how the overwhelming majority of these variants should be picked up by exome sequencing.
Figure 3: This is the old Figure 1. We hope the respected reviewer will reconsider the importance of this figure in which we show a complete list of TDVs and their breakdown by category. We now added the results of RTPCR to comply with another suggestion by the same reviewer (see below).
Figure 4: This figure summarizes the results of our reanalysis of “WES-negative” cases.
Figure S1: The new Figure S1 summarizes the results of RNA-Seq both on positive controls as well as those that were unknown.
Response: These are helpful changes. However, the authors seem to refer to figure 3 instead of figure 4 and vice-versa. There are also two entries for “Figure 2” in the figure legend.

6) The concept or transcript-deleterious variant, which is central to the paper, is unclear. The abstract defines those variants as "variants likely to cause their phenotypes by interfering with the normal transcription of the respective gene". This is wrong. Transcription refers to RNA synthesis. In contrast, the variants considered by the authors include variants affecting RNA processing (in particular splicing). Literally, a "transcript-deleterious variant" should refer to any variant deleterious for the transcript. However, they excluded protein-truncating coding variants (according to the abstract) although these variants potentially affect RNA stability by triggering non-sense mediated decay. Moreover, the variants are defined based on in silico criteria. Should not they be therefore designated as "potentially deleterious"? An alternative definition could be "transcript-encoded variants" but would then variants located outside the processed RNA such as intronic variants be considered? Without a clear conceptual definition of this central notion, the study falls apart.
We respectfully disagree with the reviewer’s assertion that we did not provide a clear conceptual definition of what transcript-deleterious variants (TDVs) are. Each class was meticulously defined in the Materials and Methods and the numerous examples of each category as well as their empirically tested RNA outcome are listed in extensive tables. As for the semantics of “transcription” vs “splicing”, we now change the overall definition from “variants likely to cause their phenotypes by interfering with the normal transcription of the respective gene” to “variants likely to cause their phenotypes by interfering with the final transcript of the respective gene” to satisfy all categories. As for logic behind excluding nonsense variants even though they may exert their effect at the level of the final transcript via NMD, we now make this clear in Materials and Methods as follows: “Although nonsense variants may exert their pathogenic effect at the level of the final transcript via NMD, we have chosen to exclude them because it is very difficult to disentangle their effect on protein from that on RNA”.
Response: Instead of “variants likely to cause their phenotypes by interfering with the final transcript of the respective gene”, what about “variants likely to cause their phenotypes by affecting the sequence or abundance of the final transcript of the respective gene”?  This definition should be provided at the first instance i.e. in the Introduction. Moreover, the introduction is confusing as it explicitly takes NMD as an example of mechanisms for transcript deleterious variants (line 37).

7) The executive definition of the transcript-deleterious variants is also unclear. In particular, what are "noncanonical splice site intronic variants" (Class c)? Also, which algorithm has been used to identify "promoter/enhancer elements" (Class f)?
We had hoped that by defining “canonical” for class a (the first and last 2bp of each intron), we do not have to define “non-canonical” for class c and avoid redundancy. To honor the respected reviewer’s suggestion, we now define class c as “non-canonical splice site intronic variants i.e. other than the first and last 2bp of an intron”. We did not use any algorithm in defining promoters and enhancers because all the identified variants therein were called based on previously established and published promoters and enhancers in the literature.
Response: The question is how far in the intron are you considering a splice intronic variant? Anywhere in the intron or according to Ensembl VEP criterion (8 bp intronic)? Moreover, a reference for the promoter and enhancer list is needed for others to reproduce class f.

12) Based on what criteria "a likely causal variant was identified in 2,429 of these families"? How is the functional relationship between genotype and phenotype assessed? In fact, there are only 156 families where Homozygosity track leads to a single locus, it is thus unclear how "likely causal" the variants of these 2,429 families are.
We are a bit perplexed by both comments. Calling a likely causal variant has been fully standardized in recent years thanks to the ACMG guidelines which we do cite in our Materials and Methods. Similarly, one need not perform any mapping at all, let alone mapping to a single critical locus, to call a likely causal variant as such.
Response : To avoid this confusion, which could happen to other readers (especially since the readership of Genome Biology is broad) an explicit reference to Material and methods should be done when mentioning “likely causal”. A short description of the ACMG guidelines principle for classifying a variant as likely causal should be given in Materials and methods. It should be made clear how the supplementary tables support the classification according to the ACMG guidelines.

Moreover, ~95% of families are consanguineous. Therefore, it may seem surprising that out of 2,429, only 156 mapped to a single locus. Could the authors comment on this point? How is the typical lack of sensitivity of the Homozygosity track mapping?


14) The second criterion for a gene to be considered as an RNA candidate is that it must have the lowest alpha_g value out of all the other patients. Therefore, each gene can be an outlier of at most one patient, which even in rare diseases is not true. It would be good to know how many candidates were found in total.
The respected reviewer is absolutely correct in that this filtering cannot be applied in a general setting. However, the reason why we applied this filtering criterion in our specific case is because we had in total 11 RNA-Seq datasets (4 in fibroblasts and 7 in LCL) from RNA samples with RIN > 8.5. These 11 cases exhibited different disease phenotypes, which allowed us to assume that the causal genes are different among them. And it is because of this small number of RNA-Seq datasets that we were able to use this very strict filtering criterion. The number of candidate genes selected prior to applying the positional-based filtering is shown in the Results section. Our alpha/beta-based filtering was able to reduce the search space from ~19,000 genes to ~100 genes in most cases.
To alleviate potential confusions, we have added the following sentence right after the statement for criterion 2: “Note that criterion 2 is based on 11 RNA-Seq datasets from RNA samples with RIN > 8.5 (4 from fibroblasts and 7 from LCL) and that this criterion was set specifically for comparison based on a small number of patients.” We also corrected a typographical error in criterion 2 and changed alpha_g to omega_g.
Response: The statement “Note that criterion 2 is based on 11 RNA-Seq datasets from RNA samples with RIN > 8.5 (4 from fibroblasts and 7 from LCL) and that this criterion was set specifically for comparison based on a small number of patients” is not found in the revised text.


16) There is no data or quantification provided for the RT-PCR measurements. A figure providing the number of samples and results would be helpful. The abstract states that definitive results from RT-PCR could be obtained for 83.4% variants, but this is not shown in Results.
Excellent suggestion! Figure 3 has been prepared accordingly. We also update Results to indicate the percentage of samples for which RTPCR provided definitive results.
Response: Figure 3 does not contain the results of RT-PCR but Figure 4. A few sentences in result must explain how the authors calculate the number 83.4% which is mentioned in the abstract. This cannot be traced now. Please point to the exact changes (line numbers) in your response.


17) The discussion states that the autozygome coordinates filter reduces the search space by a factor of 300. However, this number is never mentioned throughout the manuscript. Moreover, expression or splicing outliers detection followed by false discovery rate cutoffs give a handful of candidates genes on RNA-seq and thus this filter might not be needed with state-of-the-art methods (see Kremer et al, Nat communs. 2017; Brechtmann et al. AJHG 2018 -- there is also a recent preprint Mertes et al. biorxiv 2019).
Regarding the question about the reduction of search space by a factor of 300, we believe the respected reviewer is referring to the following sentence in the Discussion section: “For example, we show in this study that the use of autozygome coordinates drastically reduces the search space in RNA-Seq by up to a factor of 300.” This number was based on the results of 15DG2154, and it was mentioned in the subsection called RNA as a Tool to Solve “Negative” WES Cases as follows: “The RNA-Seq-based prediction generated 324 candidates. Among them, DONSON was ranked 200th on the alpha score and 281st on the beta score. With the autozygome coordinate-based filtering, DONSON was found to be the only candidate.”
That is, with the alpha/beta-based filtering, we had a list of 324 candidate genes. And with the positional filtering, we were able to correctly pick DONSON as the only candidate. Thus, we were able to reduce the search space by up to a factor of 300.
To address the other point that the respected reviewer mentioned in this comment, we would like to first reemphasize that the aim of the RNA-Seq-based analysis in the present study is to empirically test our hypothetical diagnostic yield of RNA-Seq. And this requires the use of positional filtering based on autozygome coordinates as we discussed in the manuscript. Although we are developing a diagnostic tool which integrates heterogenous data, it is out of scope to comprehensively evaluate the accuracy performance of various computational diagnostics tools in this study. Nevertheless, we have compared our alpha/beta-based approach with existing RNA-Seq-based approaches including the splicing outlier detection approach by Cumming et al. and OUTRIDER which was developed by Brechtmann et al. We found that, although OUTRIDER generated very small lists of candidate genes, as the respected reviewers predicted, it was only able to correctly predict one case out of six cases. As for performance results of the splicing outlier detection method by Kremer et al., on the contrary to the respected reviewer’s prediction, we found that this approach generated a very large number of candidate genes, suggesting that it would benefit from the use of additional sets of filtering.
Response: Thanks for clarifying. The number 300 is based on one single example and this should be emphasized explicitly in the Discussion.
We understand the focus is not on RNA-seq analysis. Hence, we strongly suggest leaving the benchmark of RNA-seq methods to another, method-oriented, paper.


18) For reproducibility, the source code should be shared. Most crucial is the source code to classify variants in either of the categories a-f.
We have uploaded the source code as well as necessary datasets to reproduce our RNA-Seq-based analysis results to GitHub (https://github.com/hkuwahara/RNASeqCausalGenePrediction). The classification of variants was manually done using our in-house database.
Response: The code to compute alpha and beta is missing. Moreover, the code showing how the different outlier tools (summarized in Table S6) were applied is also missing. I strongly suggest uncoupling this paper from the alpha/beta paper.
The authors shall describe the manual classification protocol in a way that others could reproduce it. I understand that the variants are stored in a database with proprietary format. Class a-e can be reproduced easily by others with different backends. However to reproduce the classification into class f), the list of promoters and enhancers should be provided.

22) P.19 "Our results show that only 9.5% of these genes were not expressed in either RNA source." This claim should be substantiated with the number of genes reported per tissue, Venn diagram, and Method details provided. It feels rather low but it is all about a threshold issue. This statement is representative of the entire manuscript where figures are provided without point of reference, ways to assess the reliability of the number, details in the procedure, and comparison with the literature. Also, this falls short. Investigation of GTEx tissues could corroborate further this hypothesis. Have these genes or exons indeed some tissue-specificity? If so, is the tissue / the affected tissue or related to the phenotype?
While our paper was being reviewed, a new paper just came out that is in full agreement with our conclusion that the overwhelming majority of neurodevelopmental genes are indeed expressed in blood derived cell lines (see PMID: 31911672). Thus, we do not believe that our result is an outlier. It is not clear to us what the respected reviewer meant by “this claim should be substantiated with the number of genes reported per tissue” because every single variant that we tested is listed in table S3 along with all the details concerning the source of RNA and the result of RTPCR. Furthermore, we now show that GTEx is an agreement with the 9.5% of genes not being expressed in blood or skin. Mapping individual exons for tissue-specific expression is beyond the scope of this work.
Response:  The authors must describe the criteria used to determine if a gene is expressed or not. Also, ‘in either source of RNA’ aggregates information. It would be better to know the number of genes that were not expressed on each tissue separately and jointly, hence my suggestion for a Venn diagram.

23) Abstract and p. 7: the term "premature truncating coding variants" does not exist. It is either "protein-truncating variant" or "premature termination codon".
Nonsense variants are indeed prematurely truncating coding variants. Nonetheless, we honor the respected reviewer’s request and change to “protein-truncating variants”.
Response: I could not find “protein-truncating variants”. Did the authors just delete the term?

28) P. 13 "Table S3 shows that many of these cases harbored a likely deleterious variant in a gene that was novel at the time". What does "the gene was novel" mean? Not known? Not annotated? Not associated with any disease? Not associated with this disease?
It is a standard nomenclature in human genetics to refer to a gene with no established disease phenotype as novel. The message here is that the causal variant was missed because the gene was still novel i.e. not known to be associated with a Mendelian disease (GWAS associations don’t count in this context) at the time of clinical WES. This is now clarified in the table legend to avoid confusion.
Response: The audience of Genome Biology is not human genetics but genomics. ‘Novel’ could also mean that the gene is not annotated.

29) P. 18 "Class b should be lumped with class a": there is probably a selection bias if the variants were selected to associate with the disease. Actually, effects of splice dinucleotides variants (which define splice sites) on splicing are stronger than effects of variants on the two flanking nucleotides (which have a strong quantitative effect but are not splice site defining).
We are perplexed by the suggestion to lump classes a and b together. These are very different classes from a clinical molecular laboratory perspective and combining them is not any more logical than combining other two categories of TDVs.
Response: I am as well perplexed by the suggestion of merging the two classes. This is not my suggestion but something that the authors wrote at the end of the ‘The Role of In Silico Prediction’ section, p.36 line 34: “In agreement with our suggestion that class (b) variants should be lumped with class (a)....”.

**Authors Response**

**Point-by-point responses to the reviewers’ comments:**

Reviewer #2: We thank the respected reviewer for their time and valuable input and are pleased to learn that they found our responses satisfactory. We are also pleased to read such an encouraging comment that our paper “makes a good contribution to the field providing a potential upper bound for mendelian diagnoses of TDVs with RNAseq in autosomal recessive diseases”.

Below is our response to the outstanding minor concerns:

1- The authors have done a great job on improving their manuscript. I have gone through both mine and Reviewer 1's comments and feel that they were appropriately addressed as well as the changes to the manuscript. I will note that some changes in the manuscript looked like there might be repetitive text but this might be an issue with track changes. For example "To visualize splicing events, BAM files were first converted into the hg19 coordinate using CrossMap [30] and Integrative Genomic Viewer [31] was used. We have also attempted to compare our method to previously published methods as explained in Supplemental file 1.To visualize splicing events, BAM files were first converted into the hg19 coordinate using CrossMap [27] and Integrative Genomic Viewer [28] was used."

*Response: This was indeed a track change issue and will disappear as soon as the changes are accepted (or if “simple markup” option is used).*

2- Furthermore, some of the reference changes Reviewer 1 requested I did not see changed in the new reference list. I am ok though with the authors not adding these intro papers or feeling the need to cite other RNA-seq diagnostic papers that have come out more recently. There are indeed several more beyond Reviewer 1's set where gene expression has aided a Mendelian diagnosis and a comprehensive review doesn't seem necessary here (or indeed the authors could just cite a review of this area along with their current citations).

*Response: As per the respected reviewer’s excellent suggestion, we now cite the following paper as a review to avoid additional citations: Wai, H., Douglas, A. G. L., Baralle, D. (2019). RNA splicing analysis in genomic medicine. Int. J. Biochem. Cell Biol. 108, 61–71.*

3- Minor comment: Please add a breakdown of disease categories (neuro, immune, cancer, etc) to a main figure.

*Response: We have indeed included the phenotype for each of the clinical WESnegative cases in Table 1 and Table S4. We believe that adding a breakdown of the phenotypes to the main figures will distract the readers from the main message with very little gain since the overwhelming majority of the disease categories involve neurological manifestations as we clearly state in the text.*

*With many thanks!*

Reviewer #3: We thank the respected reviewer for their valuable feedback and are pleased to see that they think that we “have addressed the core issue”, that “The manuscript is indeed much better organized now”, “The new title reflects the aim of the study in a clearer way”, “The abstract and the new figures are very helpful”.

Below is our response to the outstanding minor concerns:

1- Because the focus is not on RNA-seq analysis we strongly suggest the authors to uncouple this paper from their method paper on biorxiv, by ensuring that the main message holds independently of the RNA-seq analysis method.

*Response: We appreciate the suggestion. However, as the respected reviewer knows, we were very careful in the revised submission to present a message that stands alone independently of the RNA-Seq analysis method. Indeed, the changes in the title, abstract, text organization and even figures will leave little room for readers to think that this is an RNA-Seq paper. We maintain that the very minor section on RNA-seq is an appropriate part of a paper of this scope and would like to retain it.*

2- I acknowledge that the core idea of the manuscript is interesting, useful, and that the size of the dataset is impressive. However, I remain with the uncomfortable impression of a lack of rigour as reflected by the lack of coherence between the response and the manuscript and the limited possibilities for readers to reproduce the results and methods.

*Response: We are a bit unclear as to what exactly the respected reviewer is referring to by “the lack of coherence between the response and the manuscript and the limited possibilities for readers to reproduce the results and methods” since this was precisely the guiding principle behind all the changes we made in the revised manuscript. We assume that this ambiguous and general statement is meant as an introduction to the specific pending minor changes, which we now address fully (see below).*

3- Abstract “At least 50% of patients remain undiagnosed”. Which patients should be specified, probably “At least 50% of patients with a suspected Mendelian disorder”.

*Response: changed as suggested.*

4- Figure 4 is referred to before Figure 2. Also, Figure S2 is mentioned before Figure S1.

*Response: thank you very much for the suggestion. In the sentence “272 (15%) of which represent TDVs (Figure 4, Table S1)” we should have only cited Table S1 in order to keep the citation to Figure 4 in the correct chronological order as we now do. For Figures S1 and S2, however, we had to switch their labels in the revised submission to maintain the structure of the relevant paragraph.*

5- These are helpful changes. However, the authors seem to refer to figure 3 instead of figure 4 and vice-versa. There are also two entries for “Figure 2” in the figure legend.

*Response: this seems to be related to an issue with the way the respected reviewer viewed track changes, because there is only one entry for Figure 2 in the figure legend.*

6- Instead of “variants likely to cause their phenotypes by interfering with the final transcript of the respective gene”, what about “variants likely to cause their phenotypes by affecting the sequence or abundance of the final transcript of the respective gene”? This definition should be provided at the first instance i.e. in the Introduction. Moreover, the introduction is confusing as it explicitly takes NMD as an example of mechanisms for transcript deleterious variants (line 37).

*Response: again, there seems to be an issue with the way the respected reviewer viewed track changes because we have indeed deleted the cited entry completely in the previous revision to avoid confusion. Instead, we simply define TDVs based on very clearly defined categories. As for variants that cause NMD, we have extensively addressed this issue in the previous response. Briefly, while we acknowledge that premature truncating variants can be considered TDV by mediating NMD, we opted not to include them because it is very hard to disentangle their effect on the protein and RNA. This limitation was already fully acknowledged in the text.*

7- The question is how far in the intron are you considering a splice intronic variant? Anywhere in the intron or according to Ensembl VEP criterion (8 bp intronic)? Moreover, a reference for the promoter and enhancer list is needed for others to reproduce class f.

*Response: We made it clear in the tex that we considered any intronic variant with empirical evidence that it affects splicing. Obviously, if the variant is >50bp deep, there is a much lower chance of detection by WES and targeted RTPCR of the candidate gene (either based on a specific phenotype or by positional mapping approaches as shown in several examples in our paper) will be needed. As we indicated in our prior response letter, we did not report any novel promoter or enhancer TDVs i.e. these were all previously published and their citations are included in the respective tables.*

8- To avoid this confusion, which could happen to other readers (especially since the readership of Genome Biology is broad) an explicit reference to Material and methods should be done when mentioning “likely causal”. A short description of the ACMG guidelines principle for classifying a variant as likely causal should be given in Materials and methods. It should be made clear how the supplementary tables support the classification according to the ACMG guidelines.

*Response: We have indeed referred to the ACMG guidelines in the M/M section “Variants were filtered using gnomAD and a local population database (2,379 exomes) for allele frequency of <0.001 and were interpreted by following the ACMG guidelines”. We now revise this to increase clarity as follows “Variants were filtered using gnomAD and a local population database (2,379 exomes) for allele frequency of <0.001 were interpreted by following the ACMG guidelines to determine the likely causal variants”. Please note that given the demonstrated effect of TDVs on splicing, these variants all qualify as likely pathogenic/pathogenic by ACMG so listing this for each of the variants is redundant and unnecessary, especially after the edit we highlighted above.*

9- Moreover, ~95% of families are consanguineous. Therefore, it may seem surprising that out of 2,429, only 156 mapped to a single locus. Could the authors comment on this point? How is the typical lack of sensitivity of the Homozygosity track mapping?

*Response: This is not surprising at all for those who work with positional mapping. Having the right pedigree structure and favorable recombination events to obtain a single locus is indeed only possible in a very small percentage of consanguineous families. Again, one need not map a family to a single locus to narrow the search space, but we highlighted this very special class of families because these represent the narrowest possible search space in these settings.*

10- The statement “Note that criterion 2 is based on 11 RNA-Seq datasets from RNA samples with RIN > 8.5 (4 from fibroblasts and 7 from LCL) and that this criterion was set specifically for comparison based on a small number of patients” is not found in the revised text.

*Response: we apologize for the oversight, which is now fully addressed.*

11- Figure 3 does not contain the results of RT-PCR but Figure 4. A few sentences in result must explain how the authors calculate the number 83.4% which is mentioned in the abstract. This cannot be traced now. Please point to the exact changes (line numbers) in your response.

*Response: Correct, we were referring to Figure 4. The sentence in the abstract “Definitive results from RTPCR could be obtained from blood-derived RNA for the overwhelming majority of variants (83.4%), and only a small minority (4.5%) failed analysis on both blood- and skin fibroblast-derived RNA samples (full concordance between the two sources of RNA was noted when the gene was expressed)” is based on Table S1 that details the results of RTPCR and source of RNA for each of the tested variants. The respected reviewer is correct in that this should have also been included in a sentence or two under Results as well. This is now fully addressed by the following additional sentences:*

*“We found that 84.1% (195 out of 232) of the tested genes are expressed in the bloodderived RNA, 85.8% (199 out of 232) in fibroblast-derived RNA and 90% (209 out of 232) in the renal epithelial cells-derived RNA. The majority of genes were expressed in all three sources of RNA (75.5%), while only 2.6% (6 out of 232 genes) were not expressed in any of these sources.”*

12- Thanks for clarifying. The number 300 is based on one single example and this should be emphasized explicitly in the Discussion. We understand the focus is not on RNA-seq analysis. Hence, we strongly suggest leaving the benchmark of RNA-seq methods to another, method-oriented, paper.

*Response: We maintain that the statement in Discussion “For example, we show in this study that the use of autozygome coordinates drastically reduces the search space in RNA-Seq by up to a factor of 300” is accurate since we did use the qualifier “up to”. However, to honor the respected reviewer’s suggestion we now revise it as follows: “For example, we show in this study that the use of autozygome coordinates drastically reduces the search space in RNA-Seq (up to a factor of 300 in one case).”*

13- The code to compute alpha and beta is missing. Moreover, the code showing how the different outlier tools (summarized in Table S6) were applied is also missing. I strongly suggest uncoupling this paper from the alpha/beta paper.

*Response: We understand the respected reviewer’s preference, but removing any reference to the methodology of RNA-Seq in our paper will be a major omission in our opinion. As for the code, this is now deposited in Kuwahara H. RNA-seq-base causal gene prediction with alpha and beta scoring (Version 0.11). Zenodo. http://doi.org/10.5281/zenodo.3757089. Accessed 19 April 2020, as recommended by the journal.*

14- The authors shall describe the manual classification protocol in a way that others could reproduce it. I understand that the variants are stored in a database with proprietary format. Class a-e can be reproduced easily by others with different backends. However to reproduce the classification into class f), the list of promoters and enhancers should be provided.

*Response: Again, we have not described any novel enhancers or promoters as these were all previously published and are cited accordingly in our paper. All the variants are included in our tables and can easily be verified by others. Avoidance of description of the manual curation of the variants is exactly why we are referring to the ACMG classification so our interpretation can easily be verified by others.*

15- The authors must describe the criteria used to determine if a gene is expressed or not. Also, ‘in either source of RNA’ aggregates information. It would be better to know the number of genes that were not expressed on each tissue separately and jointly, hence my suggestion for a Venn diagram.

*Response: We now clarify in Methods that “RTPCR followed a standard number of 35 cycles and 2000ng of RNA as a template. If this standard protocol resulted in a visible band on a gel, the gene was considered “expressed”. If additional cycles or higher amount of RNA were needed, the gene was considered “poorly expressed”, otherwise, the gene was labeled as “not expressed”. Instead of a Venn diagram, we now follow the previous suggestion by the reviewer to explicitly state in Results the number of percentage of genes expressed in each tissue separately and jointly as follows: “We found that 84.1% (195 out of 232) of the tested genes are expressed in the blood-derived RNA, 85.8% (199 out of 232) in fibroblast-derived RNA and 90% (209 out of 232) in the renal epithelial cells-derived RNA. The majority of genes were expressed in all three sources of RNA (75.5%), while only 2.6% (6 out of 232 genes) were not expressed in any of these sources.”*

16- Abstract and p. 7: I could not find “protein-truncating variants”. Did the authors just delete the term?

*Response: we apologize for the oversight, which is now fully addressed.*

17- The audience of Genome Biology is not human genetics but genomics. ‘Novel’ could also mean that the gene is not annotated.

*Response: we now explicitly state that “novel” means that no OMIM disease had been assigned.*

18- I am as well perplexed by the suggestion of merging the two classes. This is not my suggestion but something that the authors wrote at the end of the ‘The Role of In Silico Prediction’ section, p.36 line 34: “In agreement with our suggestion that class (b) variants should be lumped with class (a)....”.

*Response: We believe this is a simple misunderstanding. What we meant to state is that while ACMG only classifies a as canonical splicing, we believe that b should be as well. To avoid confusion, we now edit the sentence to read “In agreement with our suggestion that class (b) variants should be lumped with class (a) (for the purpose of assigning a canonical splicing score on the ACMG classification)”.*

*With many thanks!*
